# Cyclic Tetra-Adenylate (cA_4_) Recognition by Csa3; Implications for an Integrated Class 1 CRISPR-Cas Immune Response in *Saccharolobus solfataricus*

**DOI:** 10.3390/biom11121852

**Published:** 2021-12-09

**Authors:** Alexander A. Charbonneau, Debra M. Eckert, Colin C. Gauvin, Nathanael G. Lintner, C. Martin Lawrence

**Affiliations:** 1 Department of Chemistry and Biochemistry, Montana State University, Bozeman, MT 59717, USA; alexcharbonneau94@gmail.com (A.A.C.); colin@gauvin.id (C.C.G.); nglintner@gmail.com (N.G.L.); 2 Thermal Biology Institute, Montana State University, Bozeman, MT 59717, USA; 3 School of Medicine, University of Utah, Salt Lake City, UT 84112, USA; deckert@biochem.utah.edu

**Keywords:** Csa3a, Csa3b, CARF, cyclic oligoadenylate, cOA, cyclic tetraadenylate, Cas, CRISPR-Cas, transcription factor

## Abstract

Csa3 family transcription factors are ancillary CRISPR-associated proteins composed of N-terminal CARF domains and C-terminal winged helix-turn-helix domains. The activity of Csa3 transcription factors is thought to be controlled by cyclic oligoadenyate (cOA) second messengers produced by type III CRISPR-Cas surveillance complexes. Here we show that *Saccharolobus solfataricus* Csa3a recognizes cyclic tetra-adenylate (cA_4_) and that Csa3a lacks self-regulating “ring nuclease” activity present in some other CARF domain proteins. The crystal structure of the Csa3a/cA4 complex was also determined and the structural and thermodynamic basis for cA_4_ recognition are described, as are conformational changes in Csa3a associated with cA_4_ binding. We also characterized the effect of cA_4_ on recognition of putative DNA binding sites. Csa3a binds to putative promoter sequences in a nonspecific, cooperative and cA_4_-independent manner, suggesting a more complex mode of transcriptional regulation. We conclude the Csa3a/cA_4_ interaction represents a nexus between the type I and type III CRISPR-Cas systems present in *S. solfataricus*, and discuss the role of the Csa3/cA_4_ interaction in coordinating different arms of this integrated class 1 immune system to mount a synergistic, highly orchestrated immune response.

## 1. Introduction

The basic tenets of CRISPR-Cas adaptive immunity are now well established [1,2,3]. This heritable, adaptive immune response is manifested in three distinct stages, (i) adaptation, (ii) crRNA maturation and (iii) interference. During adaptation, the CRISPR-associated proteins Cas1, Cas2 and Cas4 integrate short segments of invading DNA (protospacers) into CRISPR loci. These “spacers” are separated from those of previous invaders by a short DNA repeat, giving rise to the namesake clustered regularly interspaced short palindromic repeats, or CRISPR loci. In stage (ii), the CRISPR-locus is transcribed and the pre-CRISPR RNA is processed by Cas proteins (Cas6 or Cas12) or cellular nucleases to produce CRISPR-RNA (crRNA). In stage (iii), crRNA and Cas proteins assemble into nucleoprotein complexes that survey the cell for invading nucleic acid. Upon recognition, Cas nuclease activity then degrades the invading genetic material.

Well appreciated also is the extreme diversity of CRISPR-Cas systems. They are grouped into 2 distinct classes, Class 1 and Class 2, and further subdivided into 6 types and 24 different subtypes [4]. Relevant to the work described here are subtypes I-A, III-B and III-D belonging to class 1 systems. The *Saccharolobus solfataricus* P2 genome (formerly *Sulfolobus solfataricus* [5]) encodes three type I-A, three type III-B and one type III-D module, and harbors six CRISPR loci designated CRISPRs A–F (Figure S1) [6,7,8]. Type I-A effector complexes, also known as archaeal Cascade (aCascade), target double-stranded DNA in a PAM-dependent manner [1,9], while the type III-B and III-D effector complexes target transcriptionally active DNA and mRNA in a PAM-independent manner.

### 1.1. CARF Domains

CRISPR systems frequently include ancillary components. These include Csa3 which is associated with type I-A systems, and Csm6, Csx1 and Csx3, which are associated with type III systems [4,10,11,12]. Consistent with early bioinformatics, structural analysis of Csa3 revealed a modified dinucleotide binding domain at the N-terminus, and a MarR-like winged helix-turn-helix at the C-terminus, indicative of DNA binding [6]. The structural analysis also identified two conserved sequence motifs in the N-terminal dimerization domain [Thr-h-Gly-Phe-(Asn/Asp)-Glu-X_4_-Arg and Leu-X_2_-Gly-h-Arg]. These conserved motifs are distal to the DNA-binding domains, and form a twofold symmetric pocket that spans the dimer interface. This conserved pocket exhibits the hallmarks of a regulatory ligand-binding site, suggesting Csa3 is a transcriptional regulator under allosteric control of the N-terminal domain [6]. It was suggested that the twofold symmetric pocket with hydrophobic and positively charged side chains might be complementary to signals similar to those of cyclic dinucleotides [6].

Lintner et al. also noted a similar 3D fold, quaternary structure and conserved sequence motifs at the dimer interface of Csx1/Csm6, a second class of CRISPR/Cas ancillary protein, suggesting a similar regulatory binding site in Csx1/Csm6 [6]. Ensuing bioinformatics work rechristened this modified dinucleotide binding domain in Csa3 and Cxs1/Csm6 as the CRISPR-associated Rossmann fold, or CARF domain [13,14]. Bioinformatics, structural and functional work also identified the C-terminal domains of Csx1 and Csm6 as metal-independent HEPN ribonucleases [15,16,17], suggesting the CARF domain regulatory signal would activate Csx1/Csm6 RNase activity to complement CRISPR-Cas action, or to induce cell dormancy and/or programmed cell death [13].

### 1.2. Cyclic Oligo-Adenylate Second Messengers

In subsequent work, a fourth CRISPR/Cas ancillary protein known as Csx3 was shown to be a metal-dependent exoribonuclease, and structural analysis of the Csx3/RNA cocrystal identified a 4-base RNA fragment distant from the active site [18]. Importantly, Csx3 was then recognized as a distant member of the CARF domain superfamily, and a cyclic 4-base RNA was suggested as the regulatory ligand for CARF domain proteins [19]. 

In parallel, structural studies had also identified a cyclase domain in Cas10, the large subunit of type III surveillance complexes [20]. In beautiful work, two different groups experimentally connected the dots, demonstrating that the Cas10 cyclase domains utilize ATP to synthesize cyclic tetra-adenylate (cA_4_) and hexa-adenylate (cA_6_) when the type III effector complex is bound to target RNA. They also demonstrated these signals do indeed stimulate the endonuclease activities of Csx1 and Csm6 respectively [21,22], confirming the earlier hypotheses [6,13] for Csx1 and Csm6.

### 1.3. Transcriptional Regulation by Csa3

Two Csa3 clades have been identified, 3a and 3b [23,24]. Liu et al. have shown overexpression of Csa3a in *S. islandicus* Rey15A increases transcript levels for cas1, cas2, cas4a and csa1, resulting in acquisition of new spacers [23,25]. Further, DNA foot-printing identified Csa3a binding sites immediately upstream of the cas4a/cas1/cas2 acquisition cassette and several CRISPR loci [23], suggesting Csa3a regulates expression of CRISPR loci and cas genes involved in spacer acquisition. They also identified *S. solfataricus* Sso1445, the subject of our work, as a member of the Csa3a subfamily [23].

### 1.4. Integration of Class 1 Systems

Collectively, the work above suggests the cA_4_ second message generated by type III systems may act through Csa3a to provide an integrated Class 1 response in organisms harboring both type 1-A and type III systems [3]. Specifically, production of the cA_4_ second messenger may stimulate primed acquisition of new spacers by upregulating transcription of the acquisition cassette (cas1, cas2 and cas4), and expression of the new spacers by upregulating transcription of the CRISPR loci as well [3,6,25]. Here we show that *S. solfataricus* Csa3a does indeed recognize cA_4_. We describe the structural basis for cA_4_ recognition and cA_4_-induced conformational changes in Csa3a, and characterize DNA recognition in the absence and presence of cA_4_. Overall, the role of cA_4_ and Csa3a in the regulation of CRISPR-Cas may be more complicated than previously appreciated. With regard to genome engineering, we note that greater understanding of transcriptional regulation by cyclic oligoadenylates might enable engineered transcriptional responses upon detection of specific target genes.

## 2. Materials and Methods

### 2.1. Bioinformatics

Gene annotation was obtained from NCBI RefSeq when available, or, when no annotation was available, the ORF amino acid translation was acquired through UniproUGENE, and submitted to HHPred for profile–profile sequence alignment. Identification was considered conclusive only when the E-value was <0.001 and probability was greater than 95%. In the event no conclusion could be made, genes were submitted to HHBoost machine learning server for further aid in identification, or left as unannotated [26].

Putative *S. solfataricus* Csa3a binding sites in the Cas4a (Sso1451) promoter and the leader sequences of CRISPR loci C, D and E were identified by Liu et al. in 2017 [25]. Similarly, we also analyzed putative acquisition gene promoters and CRISPR leader sequences, submitting them to the Multiple EM for Motif Elicitation server. The results were trimmed and resubmitted until only conserved bases were remaining. The conserved motif was then queried against all available fully sequenced *Sulfolobales* genomes, and identified locations were annotated locally. 

### 2.2. Csa3a Expression and Purification

Expression and purification of SsCsa3a (Sso1445) was performed in *Escherichia coli* as previously described [6,27]. Briefly, BL21 (DE3)-pRIL *E. coli* (Stratagene, San Diego, CA, USA) were transformed with the pEXP14-6×His-Sso1445 expression plasmid [6] and a single colony was used to inoculate 5 mL of LB with 100 μg/mL ampicillin and 34 μg/mL chloramphenicol and grown overnight. Then, 750 mL of ZYP-5052 autoinduction media [28] containing 100 μg/mL ampicillin and 34 μg/mL chloramphenicol was inoculated with 750 μL of the starter culture and grown at 37 °C for 16–20 h. Cells were harvested by centrifugation at 5500× *g* for 20 min, and the pellets were stored at −80 °C.

Cell pellets were thawed and resuspended in 5 mL of lysis buffer (20 mM Tris pH 8.0, 400 mM NaCl, 0.1 mM PMSF) per gram of cell pellet. Cells were lysed by passage through a French press (American Instrument Co., Inc., Silver Springs, MD, USA). The lysate was incubated at 60 °C for 20 min to denature *E. coli* proteins and clarified by centrifugation at 30,000× *g* for 30 min. The supernatant was then applied to a gravity-flow column containing a 0.5- to 1-mL bed volume of Ni-NTA agarose (Qiagen, Germantown, MD, USA). The column was washed with 8 column volumes of wash buffer (20 mM Tris, 400 mM NaCl and 10 mM imidazole, pH 8.0), and Csa3a was eluted in 10 mM Tris (pH 8.0), 50 mM NaCl and 300 mM imidazole. Csa3a was then applied to a calibrated Superdex 75 10/300 GL column (Cytiva, Marlborough, MA, USA) equilibrated with SEC Buffer (10 mM Tris and 50 mM NaCl pH 8.0) and fractions containing the major peak were combined. Protein concentrations were determined by Bradford assay [29] using protein assay reagent (Bio-Rad, Hercules, CA, USA) and bovine serum albumin as a standard. The purity and molecular weight of Csa3a were confirmed by SDS-PAGE.

### 2.3. Isothermal Titration Calorimetry

Protein samples were prepared by passage over a Superdex 75 as described above. Lyophilized cA_4_ was resuspended in the same SEC buffer used to prepare Csa3. These Csa3a and cA_4_ stocks were centrifuged at 17,000× *g* for 10 min to remove any aggregates, and the UV absorbance of the samples was measured to determine concentrations (ε_280nm_ for Csa3a = 8960 M^−1^ cm^−1^ and ε_259nm_ for cA_4_ = 54,000 M^−1^ cm^−1^), with samples prepared by dilution into the same buffer (25 µm Csa3a dimer, 250 µM or 500 µM cA_4_). In a MicroCal ITC200 (Malvern Panalytical, Malvern, UK), 250 µM or 500 µM cA_4_ was injected (19 injections of 2 μL at 180 s intervals) into a cell chamber containing 207 μL of Csa3a dimer (25 μM). Isotherms were fit to a single-site model with ORIGIN software (MicroCal, Northhampton, MA, USA), assuming a single cA_4_ binding site in each Csa3a dimer. Titrations were performed in duplicate. The averaged results are reported in Table S1.

### 2.4. Cyclic Oligonucleotide Phosphodiesterase (Ring Nuclease) Assays

Ring nuclease assays were performed largely as previously described [30]. Briefly, unlabeled cA_4_ was added to a final concentration of 150 µM to reactions containing 10 µM SsCsa3a in 10 mM Tris, 50 mM NaCl, pH 8.0, plus SUPERase•In inhibitor and incubated at 50 °C. Then, 10 µL aliquots were removed from the reaction mixture and quenched with equal volume phenol-chloroform at desired time points. Upon quenching, 20 µL H_2_O was added to each aliquot to bring the sample volume up to 30 µL aqueous and 10 µL organic; 20 µL of the aqueous portion was then removed and dried in a SpeedVac for 30 min. Samples were then resuspended in 20 µL methanol, centrifuged at 16,000× *g* for 10 min, and the top 15 µL was removed and again dried in a SpeedVac for 30 min. Finally, samples were resuspended in 5 µL methanol, and spotted onto a glass-backed F254 silica gel TLC plate. The TLC plate was developed in 70% ethanol and 0.2 M ammonium bicarbonate (pH 9.3) until the mobile phase reached approximately 75% of the plate height. Plates were then dried and imaged with an F254 lamp and imaging box. The intensities of the residual cA_4_ were quantitated using ImageJ image analysis software and normalized to time = 0. Using KDE LabPlot 2.8.2, the data were then fit to progress curves as described by Sternberg et al. [31] to determine the first order rate constants (k_obs_).

### 2.5. Crystallization and Data Collection

Purified Csa3a in SEC buffer was concentrated to 7.5 mg/mL with a 10,000 MWCO Amicon Ultra™ spin concentrator (MilliporeSigma, Burlington, MA, USA). The protein was co-crystallized with cA_4_ using hanging drop vapor diffusion. Drops were set up at 22 °C using 2 μL of Csa3, 2 μL of well solution (14–22% PEG MME 550, 100 mM imidazole pH 7.0 and 150 mM malate at pH 7.2), and 0.2 uL of 10 mM cA_4_ (c-tetraAMP, BioLog Lifescience Institute, Bremen, Germany). Co-crystals of Csa3a with cA_4_ up to 0.30 mm × 0.075 mm × 0.075 mm in size were obtained in 3 to 5 days. Single crystals were flash frozen in liquid nitrogen and data was collected at 160 K using our MicroMax-007 X-ray generator and Rigaku R-axis 4^++^ image plate detector. Csa3-cA_4_ crystals diffracted to 2.45 Å resolution. Data were indexed, integrated and scaled in space group P2_1_2_1_2_1_ (a = 46.1 Å, b = 93.4 Å, c = 105.2 Å) using the HKL2000 software package [32]. Data quality parameters are presented in Table 1.

### 2.6. Structure Determination and Refinement

The Phaser-MR module [33] of Phenix [34] was used to determine initial phases for Csa3a in complex with cA_4_ by molecular replacement. The structure of apo-Csa3a (PDB ID: 2WTE) [6] was used as a search model. Anticipating conformational changes upon cA_4_-binding, the N- and C-terminal domains were placed separately. Two copies of each domain were placed, yielding a single dimer within the asymmetric unit (40.5% solvent). The molecular replacement solution yielded a refined LLG of 2211, a TFZ score of 20.3 and an R-value of 42.4%. The structure was completed by iterative model building with Coot [35] and refinement with phenix.refine [34]. Temperature/libration/screw parameters [36] were included in the refinement, with each of the two Csa3a chains and the single cA_4_ ligand divided into twelve temperature/libration/screw groups (chain A: 1–110, 111–157, 158–177, 178–112; chain B: 1–28, 29–55, 56–84, 85–97, 98–127, 128–157, 158–185, 186–212; cA_4_: 1–4). The final R_work_ and R_free_ values were 20.3% and 24.30%, respectively. MolProbity was used for model validation [37], with 96% of residues falling in the most favored regions of the Ramachandran plot and none in disallowed regions. The overall MolProbity score [37] was 1.51, placing Csa3a-cA_4_ in the 99th percentile for overall geometric quality among protein crystal structures of similar resolution (2.458 ± 0.25 Å). Residue numbers in the model were consistent with the native, nontagged protein sequence. Residues 160–162, 174–176, 192–195 and 213–237 of chain A, and 213–237 of Chain B were not modeled due to lack of interpretable electron density. Additional details on model refinement and model quality are presented in Table 2. The model has been deposited in the Protein Data Bank (6W11). Figures were generated in Pymol [38].

### 2.7. Electromobility Shift Assays (EMSAs)

EMSA fragments were generated by PCR using Phusion^®^ DNA Polymerase, *S. solfataricus* P2 genomic template DNA, and the locus-specific primers listed in Table S2. PCR products were the purified using the QIAquick PCR Purification Kit (Qiagen, Hilden, Germany). Then, 12 μL EMSA binding reactions containing 5.2 ng/μL DNA, varying concentrations of Csa3a, +/−10 μM cA_4_ were incubated at 37 °C for 30 min in TAE buffer. Samples were separated with a 2% *w*/*v* agarose gel, and bands were visualized via ethidium bromide staining and imaged using an Alpha Innotech AlphaImager 2200 gel imager. Data were quantified using ImageJ image analysis software, where we followed the disappearance of the unbound DNA, and modeled with GraphPad Prism.

### 2.8. Fluorescence Polarization (FP) Assays

The 5′-FAM-labeled CAPPa (20 bp) and idealized CAPPa (28 bp perfect palindrome based on our CAPPa) were generated via incubation of complementary single-stranded oligonucleotides (Table S3) in 5 mM MgCl_2_ at 94 °C for 5 min followed by slow cooling to 21 °C over 3 h. The 5′-FAM-labeled 75 bp DNA fragment was generated via PCR using Phusion^®^ DNA Polymerase (NEB), PH1-16 genomic DNA as template and a 5′-FAM-tagged forward primer and untagged reverse primer (Table S3). FP assays were conducted in 384-well black plates with optical bottoms (Nunc). Samples containing 30 nM 5′-FAM-labeled DNA probe and varied concentrations of Csa3a with or without 10 μM cA_4_ were prepared in 20 mM SPG (2.5 mM succinate 8.75 mM NaH_2_PO_4_, 8.75 mM glycine), 50 mM NaCl, pH 6.5 with a total volume of 100 μL. Samples were covered to prevent evaporation and incubated at 21 °C or 50 °C for 15 min. FP readings were recorded at 21 °C or 50 °C using a SpectraMax iD5 plate reader (Molecular Devices). mP values for the samples were calculated using a G factor of 0.314. Binding data were analyzed using GraphPad Prism.

### 2.9. Sulfolobus Strains, Growth Conditions

*S. solfataricus* PH1-16 (Δ*pyrEF*) was cultured in DT media (with 20 μg/mL uracil) at 78 °C and shaken at 65 rpm. An empty pSeSD1 vector and pSeSD1 containing *S. solfataricus* P2 csa3a (pSeSD-sso1445) were transformed into PH1-16 cells via electroporation [9,39], and transformants were selected for via growth in DT media without uracil. Cultured transformants were plated on 0.7% GelRite DT media plates and incubated at 78 °C until colony formation. A monoculture of transformed cells was started via inoculation of DT media with a single colony [9].

### 2.10. Acquisition Assay

Wild-type *S. solfataricus* PH1-16, PH1-16 containing an empty pSeSD vector, and PH1-16 containing the pSeSD-sso1445 vector were cultured in AT media (DT media with arabinose substituted for dextrose) until OD_650_ ~0.8. Overexpression of Csa3a was confirmed via protein purification from induced PH1-16 cells as for *E. coli* above. The first 2–5 leader proximal spacers of the CRISPR loci were PCR-amplified using Phusion^®^ DNA Polymerase (NEB), 3 μL of cell culture as the DNA template, and locus-specific primers (Table S4). PCR products were separated on a 2% agarose gel and visualized via ethidium bromide staining.

## 3. Results

### 3.1. CRISPR/Cas Systems in S. solfataricus

We began our investigation with further analysis of CRISPR-Cas systems in *S. solfataricus*. The *S. solfataricus* genome harbors six CRISPR loci (A–F) containing anywhere from 6–102 spacers, and at least seven cas gene clusters with varying levels of complexity. These include three type I-A systems, three type III-B systems and a single type III-D system (Figure S1). Two type I-A systems, denoted I-A (1) and I-A (2), are accompanied by genes for acquisition (cas1, cas2, cas4 and cas4a) and pre-crRNA processing (cas6). However, with the exception of a potential phage-derived exonuclease/cas4 present in the type III-D cluster, the remaining clusters lack acquisition and maturation genes, and presumably rely on those associated with the I-A (1) and I-A (2) systems.

Within these clusters, genes encoding CARF domain proteins are found associated only with the I-A (1) and I-A (2) systems. In the I-A (1) cluster, these are csx1, crn1 (sso1393) and an unannotated CARF protein of unknown function (sso1397). The crn1 gene encodes a clade 7 CARF domain with CRISPR ring nuclease activity [4,40]. CRISPR-ring nucleases degrade the cA_4_ signal, providing an off switch for the cyclic oligoadenylate signal [40,41,42,43]. In the case of sso1393, this CARF domain ring nuclease is fused to a winged helix-turn-helix domain, reminiscent of the CARF-wHTH of Csa3. *S. solfataricus* contains a second crn1 gene, Sso2081, 81 kb to the 5′ end of the I-A (3) cluster. The Sso2081 Crn1 ring nuclease shows greater ring nuclease activity than Sso1393, and is thought to be the principal player in cA_4_ degradation in *S. solfataricus* [40].

The I-A (2) locus harbors two additional CARF domain proteins, csa3b (sso1444) and csa3a (sso1445). csa3a is flanked on the 5′ side by CRISPR locus C, which separates csa3b (sso1444) from csa3a (sso1445) and on the 3′ side by a DDE-family transposase, an acquisition cassette (cas4, cas2, cas1 and cas4a) and CRISPR locus *D*. Additionally, csa3a is in the reverse orientation relative to CRIPR locus C and the acquisition cassette. Overall, this suggests Csa3a may regulate expression of these elements [6].

### 3.2. CRISPR-Associated Palindromes

**Figure 1:**
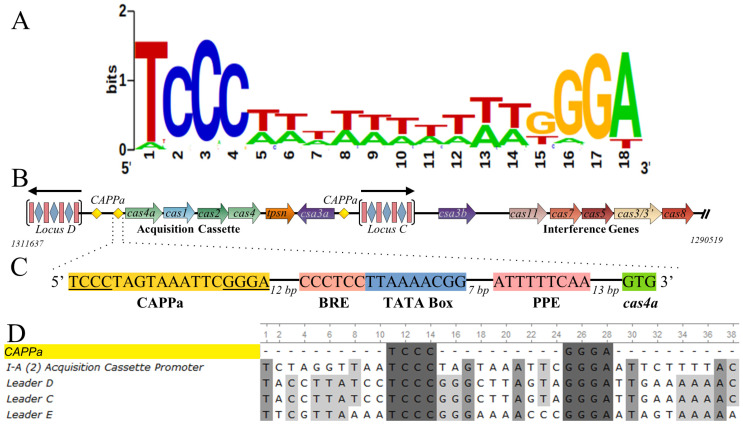
CRISPR-associated palindromes in *S. solfataricus.* (**A**). Sequence logo for the CRISPR-associated palindrome CAPPa (TCCCN_8_GGGA). (**B**) Organization of 1-A (2) acquisition cassette and adjacent CRISPR loci. CAPPa motifs (yellow diamonds) are found at four locations in the *S. solfataricus* genome; upstream of the 1-A (2) acquisition cassette as well as CRISPR loci C, D and E. (**C**) CAPPa is immediately upstream of the B recognition element (BRE), the TATA box and the promoter proximal element (PPE). (**D**) CAPPa sequences in *S. solfataricus* P2.

Relative to *S. solfataricus*, *S. islandicus* Rey15A harbors a smaller ensemble of CRISPR loci and CRISPR-associated (cas) genes. Liu et al. localized a Csa3a binding site in the promoter of the Rey15A acquisition cassette, and expanded on that in 2017 by reporting 24 bp pseudo-palindromic binding sites in the leader sequences of the two CRISPR loci as well [23,25]. They also identified similar sequences in *S. solfataricus* upstream of the I-A (2) acquisition cassette and in the leader sequences of the C, D and E CRISPR loci. Similarly, our own analysis identified these same sequences. Importantly, when we analyzed these sequences with MEME, we identified a consensus 18-bp palindromic motif that is conserved in 27 members of the order Sulfolobales (Figure 1A). Further, this motif is generally absent from the promoters of non-CRISPR-associated genes in *S. solfataricus*; i.e., it is a CRISPR-associated promoter palindrome (CAPP). To differentiate this motif from an unrelated binding site reported for Csa3b [24], we refer to this motif as CAPPa. The CAPPa element is present 12 bp upstream of the BRE and TATA box for the I-A (2)-associated acquisition genes [25], consistent with a role in transcriptional regulation of the acquisition cassette, as well as the leader sequences of CRISPR loci C, D and E (Figure 1). Last, we also find a nearly exact correspondence between the occurrence of Csa3a and the CAPPa motif in these genomes (Tables S5 and S6).

### 3.3. Csa3a Binds cA_4_

**Figure 2:**
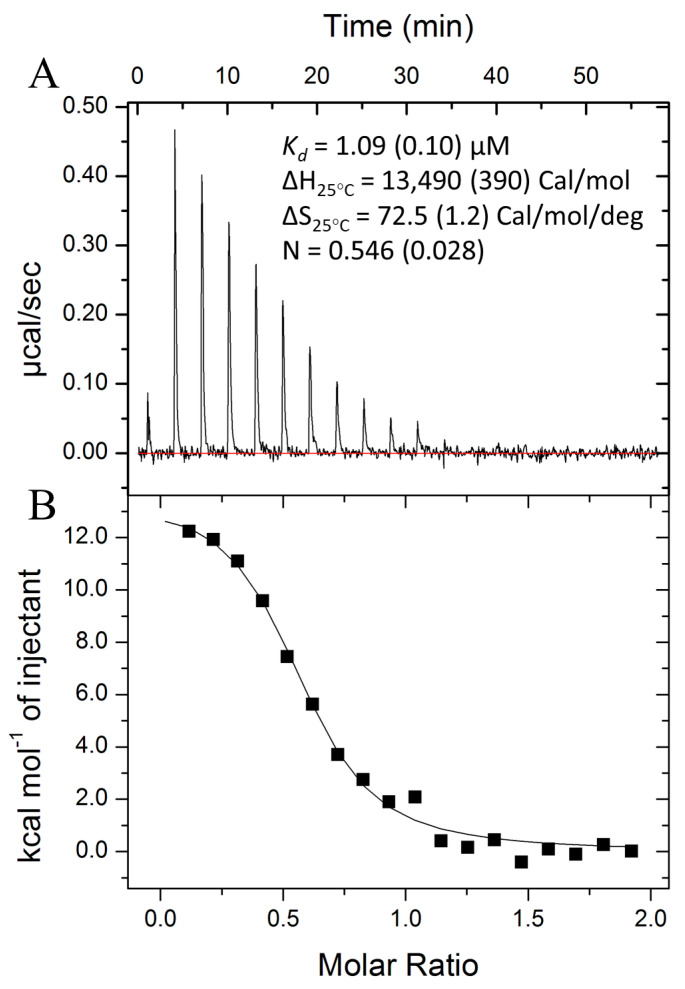
ITC analysis of the Csa3-cA_4_ interaction. (**A**) Differential power recorded over the course of the ITC experiment for titration of 250 μM cA_4_ into 25 μM Csa3a dimer. (**B**) Integration of areas under peaks corresponding to energy absorbed upon addition of cA_4_ to Csa3a as a function of molar ratio [cA_4_]/[Csa3a dimer].

We next sought to confirm the hypothetical interaction between Csa3a and cA_4_. Indeed, isothermal titration microcalorimetry (ITC) at 25 °C and 250–500 μM cA_4_ gave a dissociation constant (K_d_) of 1.0 to 1.5 μM depending on the cA_4_ concentration (Figure 2, Table S1). Notably, while the expected stoichiometry between the Csa3a dimer and cA_4_ is one to one, the ITC data show a lower than expected stoichiometry of approximately 0.6 across all titrations. However, sample centrifugation (17,000× *g*, 10 min) after ITC resulted in a distinct protein pellet, and control experiments with matching buffer in the absence of cA_4_ also showed similar pellet sizes, indicating the presence of cA_4_ does not affect the aggregation observed in the experiment. In contrast, Csa3 left at 25 °C without stirring did not give a pellet. Thus, the lower stoichiometry is likely due to protein aggregation from the agitation innate to the ITC assay. Importantly, ITC analysis also demonstrated that, at 25 °C, the binding event is endothermic, and is thus entropically driven.

### 3.4. Csa3a Lacks Ring Nuclease Activity

**Figure 3:**
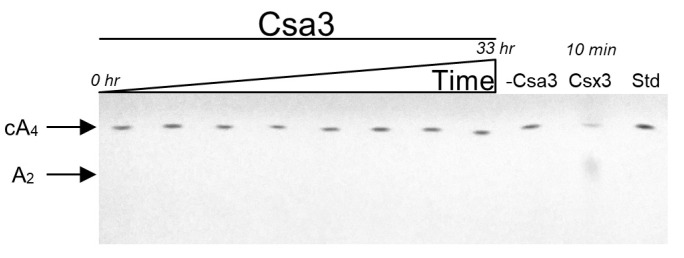
Csa3a does not exhibit ring nuclease activity. TLC analysis showing a representative time course for the degradation of unlabeled 150 µM cA_4_ incubated with 10 µM Csa3a dimer at 50 °C. Lanes 1–8 are time points at 0, 1, 2, 4, 6, 8, 16, 24 and 33 h. Lane 9 was a 33 h incubation in the absence of Csa3a. Lane 10 was a 10 min incubation with 10 µM AfCsx3 dimer and 200 µM Mn^2+^ to serve as a positive control [30]. Lane 11 was loaded with 1.5 nmol cA_4_ standard.

CARF domain proteins, like Sso1393, frequently exhibit low-level ring nuclease activity that attenuates their activation by cA_4_ [40,43,44,45]. To determine if Csa3a possesses ring nuclease activity and is potentially self-regulating, unlabeled cA_4_ was incubated with Csa3a for up to 33 h (Figure 3). We observed no cA_4_ degradation, suggesting the Csa3a CARF domain acts as a signal receptor, but is not involved in clearing the cA_4_ signal. In this regard, we note that Csa3a lacks a putative catalytic lysine residue found in many CARF ring nucleases [40].

### 3.5. Csa3a Binds cA_4_ in the CARF-Binding Pocket

To elucidate the molecular basis for cA_4_ recognition by Csa3a and any conformational changes induced by cA_4_ binding, we co-crystallized Csa3a with cA_4_ and determined the structure by X-ray crystallography. While the crystallization conditions were significantly different from those reported for the cA_4_-free structure of Csa3a first reported by Lintner et al. [6], it crystallized in the same space group with similar unit cell parameters (Table 1). Consistent with this crystal form, the asymmetric unit contains a single Csa3a homodimer, and the overall structure is largely similar to the cA_4_ free structure. However, the position of the wHTH domains relative to the CARF domains are noticeably different, and these larger conformational changes appear to be driven by subtle conformational changes in the N-terminal CARF domain associated with cA_4_ binding.

The N-terminal CARF domain retains its overall fold, a doubly wound, mixed β-sheet with flanking α-helices. Similar to a classic dinucleotide binding domain, the first five β-strands (β_1_-β_5_) run parallel and are connected by helical, righthanded crossovers. However, the fold then diverges from the classic Rossmann fold, as β_5_ makes a reverse turn into β_6_, which runs antiparallel to β_5_, giving the sheet a β_3_ (↑)-β_2_ (↑)-β_1_ (↑)-β_4_ (↑)-β_5_ (↑)-β_6_ (↓) topology. The reverse turn into β_6_ also results in the loss of a covering alpha helix. Critically, CARF domain dimerization restores this structural element, placing α_4_ of the second subunit across the β_5_/β_6_ end of the β-sheet. The α_4_ helices are thus buried at the dimer interface, where they run parallel to each other. Overall, the non-canonical β_5_/β_6_ reverse turn and loss of the connecting α-helix is critical to dimer formation, and are thus defining features of the CARF domain fold [6,30].

**Figure 4:**
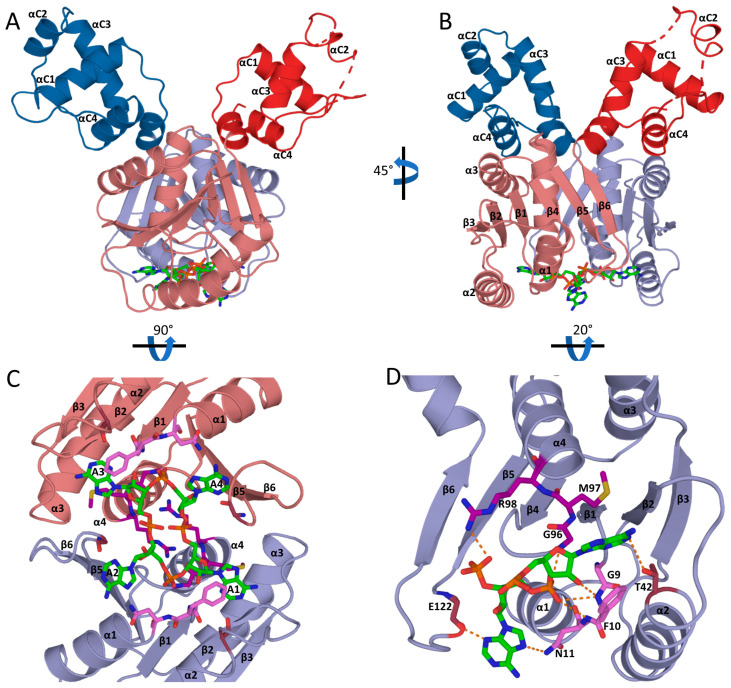
The Csa3a CARF domain binds cA_4_. (**A**) Ribbon diagram of the Csa3a dimer bound to cA_4_. The N-terminal CARF domains that form the cA_4_ binding pocket are colored light red/blue, the C-terminal wHTH domains are colored dark red/blue and cA_4_ is shown in a stick representation with C atoms colored green. A single cA4 molecule is bound in the twofold symmetric ligand binding site, across the dimer interface of the CARF domain. (**B**) The cA_4_-bound Csa3a dimer rotated 45° about the vertical axis shows the location of cA_4_ in the binding pocket relative to secondary structural elements in chain B (red). (**C**) Ribbon diagram of the cA_4_-bound Csa3a dimer looking into the cA_4_ binding pocket. Relative to A, the structure is rotated 90° about the horizontal axis. Motif 1 (pink), motif 2 (purple), T42 (deep red) and E122 (deep red) which make direct interactions with cA_4_ are represented as sticks. (**D**) Ribbon diagram of a single Csa3a subunit bound to bases 1–2 of cA_4_. The structure is rotated −20° about the horizontal axis relative to B. Hydrogen bonds are shown as orange dashes.

As expected [6], a single cA_4_ molecule is bound within a two fold symmetric pocket spanning the subunit interface at the base of the Csa3a dimer (Figure 4, Videos S1 and S2, Figure S2). The floor (or ceiling, depending on orientation) of this pocket is formed by neighboring β_4_α_4_ loops from each of the two subunits. Principal residues in this loop are Gly96 and Arg98. The Arg98 side chains run antiparallel to each other as they reach across to the subunit interface and hydrogen bond to the main chain carbonyls of Gly96 in the adjacent subunit. The unsaturated Gly96 main chain amines and the Arg98 side chains then mediate alternating interactions with the phosphate groups of cA_4_. These interactions explain the strong conservation of these residues in motif 2 of Lintner et al. [6].

cA_4_ is bound in an oval-shaped conformation with all bases present in the anti-conformation, radiating outward from the oval-shaped ribose-phosphate backbone. Further, each ribose is present in a 2′-endo conformation. Consistent with this, Molprobity [37] identifies the dihedral backbone conformations for nucleotides 2 and 4 at the distal ends of the oval as 6p conformers, and assigns nucleotides 1 and 3 as outliers of the 2o conformer [46]. Details for the RNA conformers are found in Table S7. The β_1_α_1_ and β_5_β_6_ loops project above the floor of the cA_4_ binding site in an alternating pattern about the dimer axis, where they successively interdigitate between each of the four adenosine moieties. Specifically, the β_1_α_1_ loop inserts between bases 1 and 2 in subunit A, and between bases 3 and 4 in subunit B. Similarly, the β_5_β_6_ loop in subunit A rises between bases 2 and 3, and the same loop in the B subunit between bases 1 and 4 (Figure 4).

The first nucleoside is thus bound in a groove with walls formed by the β_5_β_6_ loop of subunit B and the β_1_α_1_ loop of subunit A. The conserved Gly9-Phe10 in the β_1_α_1_ loop (motif 1 of Lintner et al. [6]) play a pivotal role. The main chain NH of Phe10 hydrogen bonds to O2′ of the ribose and the edge of the adenine base packs against the main chain of Gly9, allowing the Phe10 side chain to extend over the adenine ring. Val39 of the β2α2 loop also packs on top of the adenine ring, while the Met97 side chain (β_4_α_4_ floor loop) provides a foundation underneath the adenine to complete a hydrophobic binding pocket. Except for Thr42 in the β_2_α_2_ loop, which H-bonds to N1 of the adenine moiety, base specific interactions are not obvious.

In contrast to the first nucleoside, the second adenosine is bound entirely within subunit A, between the β_1_α_1_ and β_5_β_6_ loops. The adenine ring settles on top of main chain atoms of the β_5_β_6_ loop (residues 122–124) and Phe14 of the β_1_α_1_ loop, leaving the opposite face of the adenine solvent exposed. In addition, Thr13 accepts an H-bond from the adenine -NH_2_ while the Asn11 side chain donates a hydrogen bond to N7 of the adenine ring. Finally, the Asn11 main chain NH hydrogen bonds to the 5′phosphate of this nucleotide.

Overall, then, the first subunit plays a predominant role in recognition of the first two nucleotides of cA_4_. Similarly, the second subunit largely mediates recognition of the last two nucleotides, using a similar set of interactions. However, the cA_4_ is not bound in a completely symmetric fashion. Notably, a crystal contact with a neighboring asymmetric unit results in a cation-π interaction between Arg200 and the solvent-exposed adenine ring of the fourth nucleotide. This interaction appears to pull the adenine out of its binding pocket, breaking the interactions with Asn11 and Thr13. Further, the β_5_β_6_ loop near the crystal contact is also found in an alternate conformation relative to subunit A. Finally, each of the ribose moieties are present in a 2′-endo conformation and for residues 1, 2 and 3, the 2′-OH is hydrogen bonded to either O1P or O2P of the neighboring phosphate group. While the ribose in the 4th nucleotide is also 2′-endo, it reaches across the cA_4_ molecule to instead hydrogen bond to the O2P of the 3rd nucleotide. This is possible, in part, because the phosphate group linking the 2nd and 3rd nucleotides does not sit down on the floor of the pocket, as it does for the 5′-phosphate of the first nucleotide. Whether these differences are the result of crystallographic contacts, or there is inherent asymmetry in the solution bound form, is not clear.

### 3.6. cA_4_-Induced Conformational Changes

**Figure 5:**
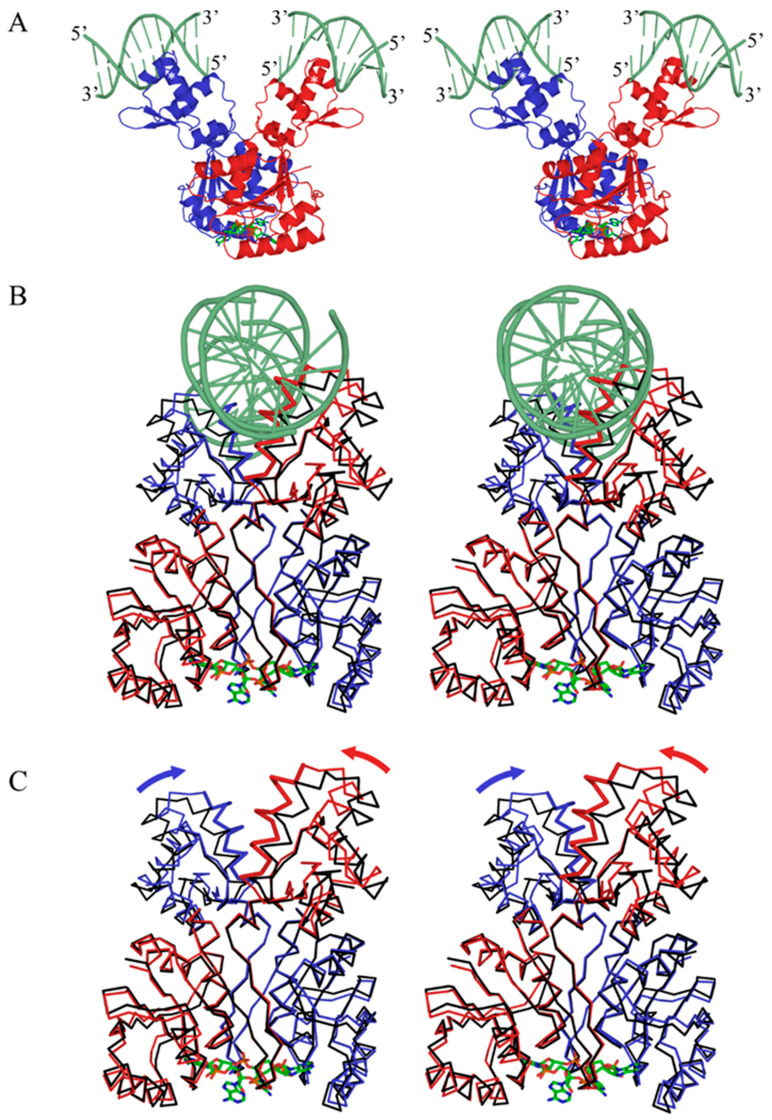
cA_4_-induced conformational changes. (**A**) Divergent stereo image of the Csa3a/cA4 complex docked to DNA (light green) by superposition of individual wHTH domains onto *B. subtilis* OhrR (PDB code: 1Z9C [47]) (RMSD: 1.7 Å). We were unable to superposition the Csa3a dimer onto a single strand of B-form DNA, suggesting further conformational changes in Csa3a or DNA bending might occur. (**B**) Significant movement of the wHTH domains is seen at the putative protein/DNA interface in the Csa3a/cA_4_ complex (blue and red) relative to the unbound structure (black). (**C**) The rotation of the wHTH domains (direction emphasized by arrows) relative to the CARF domain is a result of modest conformational changes in the CARF domain.

Local to the cA_4_ binding site, the most prominent conformational changes are modest movements of the β_1_α_1_ and β_2_α_2_ loops as they move away from the dimer axis, expanding the conserved pocket to accommodate cA_4_. At the same time, there are significant side chain rearrangements as Phe10 rotates over the adenine ring of the first (and third) nucleotide. These movements are accompanied by a small, yet significant rotation of the β_1_α_1_β_2_α_2_β_3_α_3_ end of the CARF domain (Figure 5, Video S1). Critically, the α_3_ helix runs antiparallel to the C-terminal helix of the winged helix-turn-helix DNA binding domain. This small rotation of the α_3_ helix is, in turn, transmitted to the C-terminal helix of the wHTH domain, acting as a pivot point for an approximate 7° rotation of the wHTH domain relative to the CARF domain. Relative to the Csa3a dimer, the net result is a twisting movement of the wHTH domains towards each other, into a conformation that would appear more favorable for binding linear B-form DNA. However, superpositional DNA docking using the same dimeric OhrR/DNA complex (PDB code: 1Z9C) originally used by Lintner et al. [6] suggests additional movement of the wHTH domains or some DNA bending might still be required (Figure 5, Video S2).

**Figure 6:**
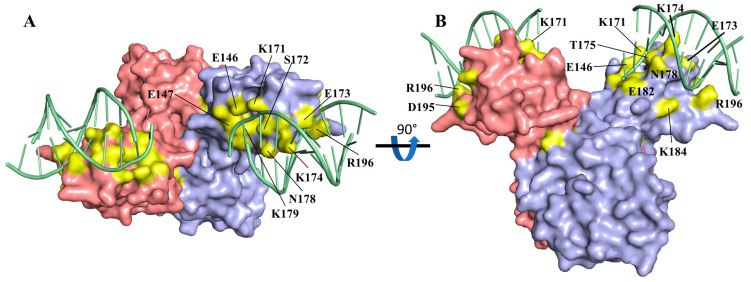
Conserved residues in Csa3a wHTH domain. (**A**) Surface model of Csa3a with strictly conserved residue in the wHTH domain in yellow and DNA in orange. (**B**) Rotated 90° about the horizontal axis. The majority of the conserved surface residues in the wHTH domain are located at the putative protein-DNA interface.

Our previous multiple sequence alignment did not differentiate between Csa3a and Csa3b [6]. While this alignment nicely identified the conserved cA_4_ binding site, considering the different DNA sequences recognized by *S. islandicus* Csa3a and Csa3b [24,25,48], it might not be expected to identify conserved residues involved in DNA recognition. We thus constructed a new multiple sequence alignment with 21 Csa3a sequences. The alignment identifies three strictly conserved residues in the recognition helix—E173, K174 and N178—that are positioned to make base-specific interactions. Additional conserved resides positioned to interact with the ribose-phosphate backbone include E146, E147, K171, S172, T175, K179, E182, K184, G193, D195 and R196 (Figure 6).

### 3.7. Csa3a Binds CAPPa Nonspecifically, in a Cooperative and cA_4_-Independent Manner

**Figure 7:**
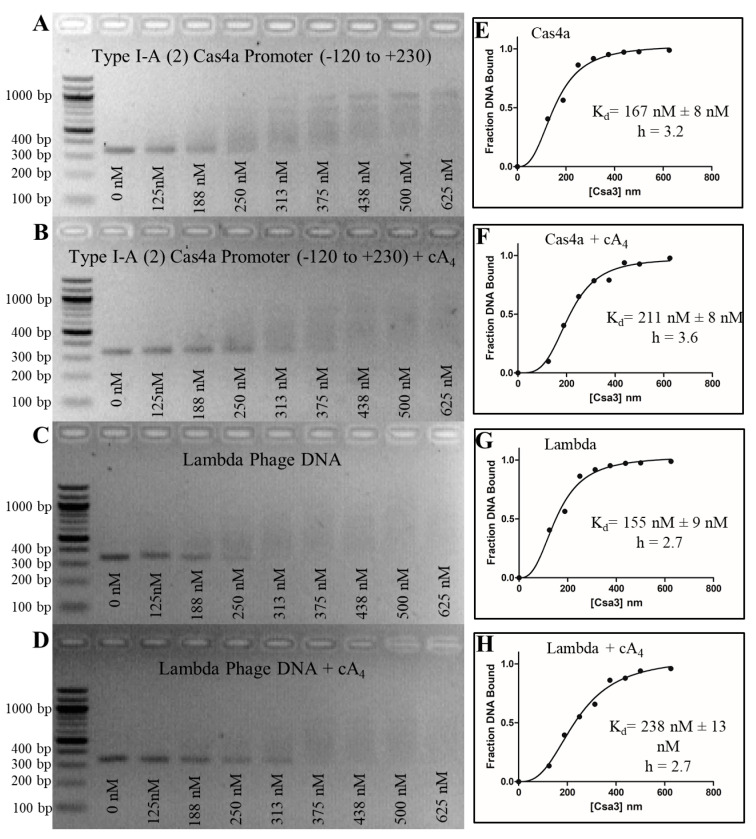
Nonspecific binding to the type I-A (2) cas4a promoter. (**A**–**D**) Csa3a binds both the cas4a promoter region containing CAPPa and the control Lambda DNA fragments with 150–250 nM affinity, indicating a nonspecific interaction in the absence of cA_4_. The addition of cA_4_ does not affect binding affinity or specificity. EMSA experiments were performed with 18 nM DNA and increasing [Csa3] dimer (denoted below the lanes). (**E**–**H**) Graphs generated in GraphPad Prism display curves for specific binding with a Hill coefficient (h) fit to binding data generated using ImageJ image analysis software. EMSA fragments are detailed in Table S4.

We first attempted to demonstrate cA_4_-modulated recognition of the putative Csa4 promoter for the *S. solfataricus* I-A (2) acquisition cassette using an electromobility shift assay (EMSA). We found that Csa3a will indeed shift a 350 bp fragment incorporating the putative promoter with relatively high affinity, giving a measured K_d_ of approximately 250–300 nM (Figure 7). Interestingly, the binding curve was sigmoidal, suggesting DNA binding was cooperative, and indeed, the fit to the observed data improved when the Hill coefficient was not constrained. Similar results were found for fragments containing the conserved CAPPa motifs in the leader sequences of CRISPR loci C, D and E as well (Figure 3, Figure 4 and Figure 5). When cA_4_ was included in the assay, however, affinity for the putative promoter region was not increased. In addition, negative controls that lacked the palindromic motif, BRE and TATA binding sites bound with similar affinity, indicating the observed DNA-binding is nonspecific. Consistent with nonspecific binding and the length of the target DNA, we observed a spectrum of shifted products, indicative of multiple binding events per DNA strand.

We considered whether the affinity of Csa3 for cA_4_ (K_d_ ~1 µM) might be insufficient for the EMSA assay. Specifically, that if cA_4_ dissociates from Csa3, it is pulled in front of the Csa3/DNA complex during electrophoresis, separating it from the complex and changing the affinity of Csa3 for DNA. We thus developed a fluorescence polarization (FP) assay in which the cA_4_ concentration remains constant. This assay also allowed us to work at temperatures as high as 50 °C, and at biologically relevant pH (pH = 6.5, [49,50]). Preliminary work at 21 °C and 50 °C indicated binding affinity and specificity were not affected by temperature. We subsequently chose to work at 50 °C, as this temperature is closer to the optimal growth temperature for *S. solfataricus* (80 °C). Additionally, changing pH from 8.0 to 6.5 did not affect binding affinity or specificity.

**Figure 8:**
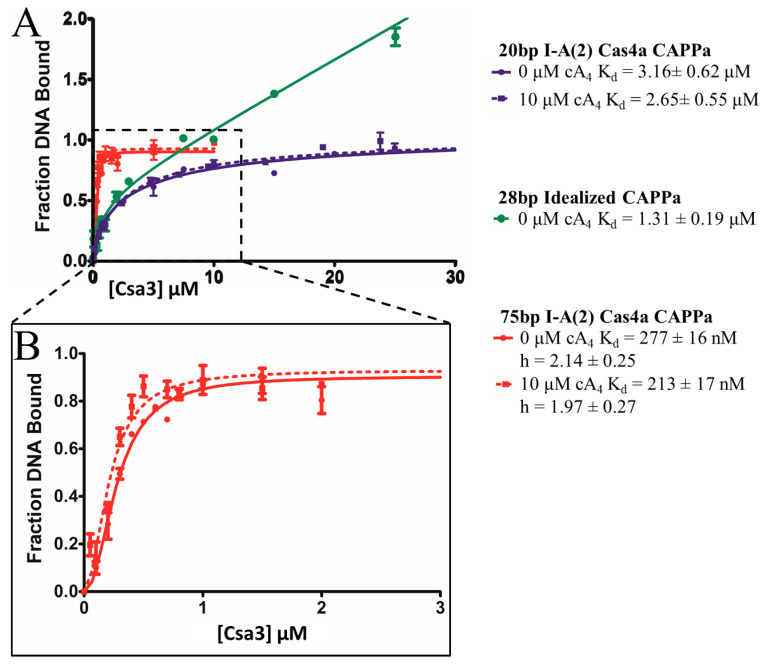
Csa3a binds CAPPa nonspecifically, in a cooperative and cA_4_-independent manner. (**A**) DNA binding was measured by fluorescence polarization at 50 °C. Both the 20 bp I-A (2) cas4a CAPPa (blue) and 28 bp idealized CAPPa (green) bound with low micromolar affinity, while the 75 bp I-A (2) cas4a CAPPa (red) was bound with 200–300 nM affinity. The addition of 10 µM cA_4_ (dotted lines) did not significantly change the affinity. Linear nonspecific binding after saturation was observed for the 28 bp idealized CAPPa. (**B**) Cooperative binding with a Hill coefficient of ~2 was observed for the 75 bp I-A (2) Cas4a CAPPa fragment. ssDNA oligonucleotides/primers used for generation of FP probes are listed in Table S4.

We first examined the affinity of Csa3a for a 20 bp fragment centered on CAPPa in the I-A (2) cas4 promoter. Surprisingly, the affinity for this smaller 20 bp fragment was only 3.2 +/− 0.6 µM, much less than for the 350 bp fragment used in the EMSA assay (Figure 8). Critically, a statistically significant increase in affinity in the presence of cA_4_ (K_d_ = 2.7 +/− 0.6 µM) was not observed. We also observed binding was no longer cooperative, which might be explained by the smaller DNA fragment (20 bp) which is able to accommodate only a single Csa3 dimer. Indeed, when we instead used a 75 bp fragment, extending in the upstream direction away from the BRE and TATA elements, cooperative binding was restored, along with the higher affinity (K_d_ = 270 +/− 20 nM) previously observed in the EMSA assays. However, cA_4_ again failed to provide a significant increase in affinity (K_d_ = 210 +/− 20 nM). We thus concluded that *S. solfataricus* Csa3a binds CAPPa nonspecifically, in a cA_4_-independent manner. This is clearly at odds with a simple model wherein cA_4_ increases the affinity of Csa3a for CAPPa, Csa3a then binds CAPPa, with subsequent recruitment of TFB, TBP and RNA polymerase. Given the data supporting transcriptional control of the acquisition cassette and CRISPR loci in *S. islandicus* [23,25], alternative models for the initial high-affinity interaction involving additional proteins might need to be considered.

In this light, the cooperative nature of the nonspecific binding remains of interest. Our modeling suggested the 20 bp fragment was sufficient to allow binding by the Csa3 dimer, but to ensure the target CAPPa sequence was indeed long enough to accommodate the full span of interactions, we repeated the experiment with a 28 bp DNA fragment centered on an idealized CAPPa motif (Table S3). This intermediate length fragment showed affinity (K_d_ = 1.3 +/−0.2 µM) close to that of the 20 bp CAPPa, again with non-cooperative binding. We did, however, observe additional low-affinity binding at higher ligand concentrations, indicative of a second, even lower-affinity binding site. This mechanism was not observed for either the 20 bp or 75 bp fragments. We rationalize this as binding of a single Csa3a subunit to DNA extending from either side of the higher-affinity Csa3/DNA complex. In this light, we conclude the nearly tenfold difference in affinity between the 20 and 75 bp targets is a result of the cooperative binding of two dimers, leading to increased affinity compared to the 20 and 28 bp fragments, which allow binding of only a single dimer.

### 3.8. Csa3a Overexpresssion Does Not Stimulate Spacer Acquisition in S. solfataricus

**Figure 9:**
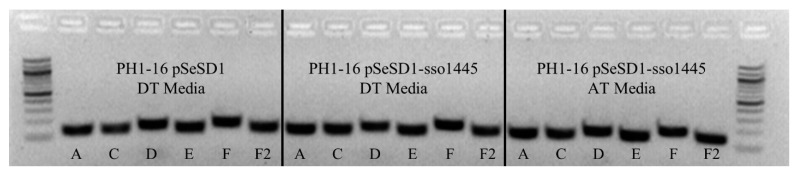
Overexpression of Csa3a in *S. solfataricus* does not lead to spacer acquisition. Leader proximal regions of the CRISPR loci were amplified from *S. solfataricus* PH1-16 cells carrying the empty pSeSD1 plasmid grown in DT media (uninduced, **left**), cells carrying the pSeSD1-sso1445 Csa3a expression plasmid grown in either DT media (uninduced, **middle**) or AT media (arabinose induced, **right**). Acquisition of a new spacer would appear as a secondary band ~60 bp larger than the band amplified from WT cells. CRISPR loci are labeled below the bands. F2 is the second half of locus F, which is separated from the leader proximal region (Figure S1).

Liu et al. demonstrated that Csa3a overexpression stimulates spacer acquisition in *S. islandicus* [23]. We duplicated this experiment in *S. solfataricus* by transforming PH1-16 cells with a pSeSD1 vector containing SsCsa3a (pSeSD-sso1445), inducing overexpression with arabinose, and PCR amplifying the leader proximal regions of the CRISPR loci. The PH1-16 cells are an *S. solfatricus* P1 ∆pyrEF variant with CRISPR-Cas gene content and organization that is highly similar to *S. solfataricus* P2. The Csa3 proteins in these strains show 100% identity at the amino acid level. Interestingly, we did not observe new spacer acquisition upon Csa3 overexpression in this second *Sulfolobales* (Figure 9). To confirm the negative result was not due to a heterogeneous culture of transformed and untransformed cells, we repeated the experiment with a monoculture started from a colony of transformed PH1-16 cells grown on DT GelRite plates with the same result. In addition, we also affinity purified the His-tagged Csa3a from these same cells, confirming that Csa3a was indeed expressed.

## 4. Discussion

### 4.1. Physiological Implications of Low-Micromolar cA_4_ Affinity

While cyclic oligoadenylate signals have been identified for a number of CARF domain proteins, including those with RNase (Csx1/Csm6) and DNase effector domains (Can1/Card1 [51]), as well as single-domain ring nucleases (Crn1, Crn3 [18,30,40]), to our knowledge this is the first formal demonstration that cA_4_ is recognized by CARF domain transcription factors with wHTH effector domains. We were initially surprised to find the affinity of Csa3a for cA_4_ is significantly lower than that reported for these other CARF domain proteins [30,51], leading us to ask if cA_4_ recognition is physiologically relevant. In this regard, Athukoralage et al. have modeled cellular cA_4_ levels over the course of viral infection [42]. Their work suggests that detection of a single viral RNA by type III systems results in a burst of cA_4_ production, potentially leading to cellular concentrations in the order of 6 µM cA_4_. In this light, 1.1 micromolar affinity for cA_4_ could indeed be physiologically relevant. It is also consistent with evidence that CRISPR systems have evolved to avoid deleterious levels of autoimmunity by limiting the rate of spacer acquisition [23], and that increased rates of spacer acquisition in mutants have been shown to lead to higher levels of toxicity [2]. The relatively low affinity of Csa3a for cA_4_ may reflect fine-tuning to balance the benefits of protection against a deleterious autoimmune response [23]. In this scenario, acquisition is a tightly regulated event, that is activated only when type III systems have detected invading mRNA, and existing type I systems have failed, leading to sustained production of cA_4_ by the type III systems.

### 4.2. cA_4_-Induced Conformational Changes

The structure of the Csa3a-cA_4_ complex is among the first CARF domain structures to show significant conformational changes upon cA_4_ binding. This may reflect the lack of ring nuclease activity. As expected, the conserved motifs of Lintner et al. [6] play significant roles in anchoring cA_4_ in the binding pocket. Local conformational changes in the CARF domain binding pocket upon cA4 recognition then propagate globally to move the wHTH domains into positions that appear more favorable for DNA recognition. Specifically, cA_4_ binding pushes on the sides of the binding pocket, causing a relative rotation of a core α_1_, β_2_, α_2_, β_3_, α_3_ unit within each CARF domain. This movement, in turn, results in a pivot of the wHTH domains about the α_3_ helix of the CARF domains, as the wHTH domains move toward each other (Videos S1 and S2). Though movements of the Csa3 effector domains are more modest, these movements are reminiscent of those seen in CARD1 [51], and collectively these structures may define features common to the activation of CARF domain proteins by cA_4_.

### 4.3. cA_4_ Recognition Is Entropy-Driven

The endothermic binding observed by ITC indicates an entropy-driven process, at least at 25 °C. Notably, the adenine bases in the cyclic oligoadenylates are solvent exposed and unable to base stack. Clathrate-like water structures are thus expected on each side of the solvent-exposed bases in the unbound ligand. Upon binding, the bases are desolvated and the ordered water is released, which is an entropically favorable process. In addition, the cyclic nature of the ligand suggests it is largely pre-ordered for binding, as is the binding pocket. Thus, there is a relatively small entropic penalty for immobilization of the ligand in the binding pocket. Overall, the increased disorder in the released solvent is greater than the increased order in the protein and ligand, resulting in an entropy-driven process for recognition of cA_4_ by Csa3. Importantly, these observations suggest entropy will play a significant role in driving cOA binding at thermophilic temperatures, and for the higher-affinity interactions of other CARF domain proteins as well.

### 4.4. Transcriptional Regulation of Spacer Acquisition and CRISPR Loci by Csa3

Evidence that Csa3a regulates expression of the acquisition genes and plays a role in regulating the uptake and expression of new spacers in *S. islandicus* is quite strong. In particular, overexpression of Csa3a in *S. islandicus* Rey 15A increases transcription of the acquisition cassette and the incorporation of new spacers, and Csa3a has been observed to bind the *S. islandicus* cas4 and cas1 promoters in vivo [22,26]. It would be surprising, then, if Csa3a were not involved in regulating spacer uptake in other members of the Sulfolobales as well, especially since there is strong synteny among type I-A (2)-like acquisition cassettes in the Sulfolobales (Table S5), and the presence or absence of Csa3a in these systems correlates nearly exactly with the presence or absence of CAPPa in the Cas4a promoters (Tables S5 and S6). At the same time, however, Csa3a overexpression in *S. solfataricus* fails to stimulate spacer acquisition, indicating distinct difference between *S. islandicus* and *S. solfataricus*. These differences are perhaps not so surprising as CRISPR-Cas in *S. solfataricus* is significantly more complex with regard to the number of cas genes and CRISPR loci. Further, in contrast to activation by Csa3a, *S. islandicus* Csa3b instead represses spacer acquisition [52]. How overexpression of Csa3a overcomes repression by endogenous Csa3b in *S. islandicus* is unknown, as is the state of cA_4_ production under these experimental conditions. The lack of spacer acquisition in *S. solfataricus* upon overexpression of Csa3a may simply indicate Csa3a is unable to counter repression by Csa3b or other regulatory elements. Clearly, critical details remain to be elucidated in both *S. islandicus* and *S. solfataricus.*

Overall, we believe transcriptional regulation by Csa3a in response to the cA_4_ second messenger remains an attractive hypothesis, one that is strengthened by observation of the cA_4_/Csa3a interaction and the associated conformational changes in the structure of Csa3a presented here. At the same time, our data rule out a simple model wherein cA_4_ produced by Cas10 in one of the type III surveillance complexes activates Csa3a binding to the CAPPa motif, followed by recruitment of transcription factor B (TFB), TATA binding protein (TBP) and RNA polymerase. A more complicated model might also be indicated by the non-canonical TATA box in the acquisition cassette promoters reported by Liu et al. [25]. Nonetheless, a specific high-affinity interaction with the promoter elements is likely to require the presence of additional factors, and to be a cooperative event.

### 4.5. An Integrated, Synergistic Class 1 CRISPR-Cas Immune Response

**Figure 10:**
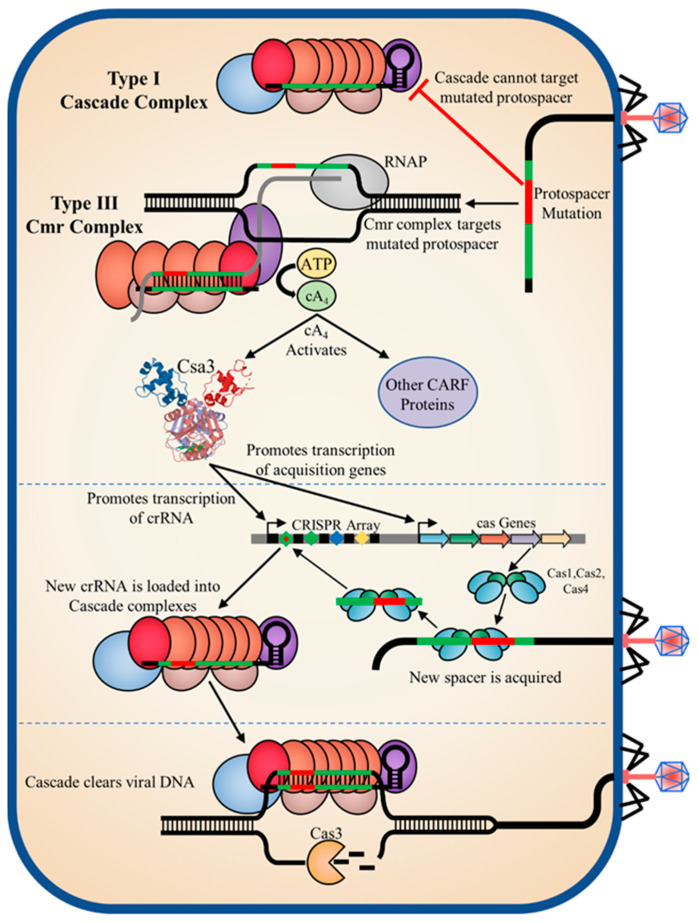
Model for an integrated Class 1 immune response. (Top) Infection with a virus harboring an inexact match to archived CRISPR spacers may hinder recognition by PAM-dependent type I systems, allowing the infection to progress to transcription of viral mRNA. The more promiscuous PAM-independent type III interference complexes may still recognize the mRNA target and produce cA_4_. cA_4_ activates Csa3a (and potentially Csa3b), which promotes transcription of acquisition genes, resulting in spacer acquisition. Csa3a also promotes transcription of new crRNAs which are loaded into aCascade surveillance complexes, restoring recognition of the invading viral DNA to the type I systems.

Regardless of the specific mechanisms of transcriptional activation and repression by Csa3a and other Csa3 family members, confirmation of cA_4_ as a Csa3a ligand in *S. solfataricus* now provides a critical, direct link between the type III surveillance complexes that synthesize cA_4_ upon detection of invading nucleic acid [53], and Csa3 family transcription factors that regulate acquisition gene expression, transcription of CRISPR loci, DNA repair and potentially the transcription and regulation of type I-A surveillance complex activity [6,23,24,25,54]. Thus, the general pathway for Csa3a-mediated spacer acquisition can still be inferred (Figure 10). For example, consider a cell infected with virus containing a protospacer mutation in the PAM sequence, rendering it untargetable by the type I-A Cascade systems. The mutated protospacer will still be targeted by the more promiscuous and PAM-independent type III surveillance complexes. Upon recognition of the mutated protospacer, the Cas10 subunit in the type III surveillance complex will synthesize cA_4_. The cA_4_ second message then activates Csa3a, inducing expression of the acquisition genes, the uptake and expression of new spacers, and their incorporation into both type I-A and type III surveillance complexes, which are then able to cure the infection. In this context, the type I-A and type III surveillance complexes are not just complementary, they are synergistic, and the integrated response provides a more robust and successful antiviral response than either of the standalone systems. In this context, the multitude of CRISPR loci and cas gene clusters in *S. solfataricus* is far more than a diverse and perhaps redundant collection of various CRISPR-Cas systems; they represent different arms of an integrated class 1 immune system. Csa3 family transcription factors and the cA_4_ second messenger lie at the heart of this system, where they coordinate a synergistic, highly orchestrated Class 1 CRISPR-Cas immune response.

### 4.6. Caveats and Additional Considerations

We cannot rule out the possibility that *S. solfataricus* Csa3a performs some cA_4_-independent functions, and that Csa3 in general may sense other ligands. While Csa3 shows strong association with type I-A systems, it is also occasionally associated with I-B, I-D III-A, III-B and III-D loci [10,14]. In addition, many genomes encoding Csa3 apparently lack Cas10, and in most Halobacteria and Methanobacteriales, Csa3 is encoded outside the CRISPR-Cas loci [14]. Indeed, Csa3 is even found in organisms apparently lacking CRISPR-Cas [6]. In these cases, it is not clear what the regulatory ligand for Csa3a might be. One exciting possibility, however, is the cyclic-oligonucleotide-based second messengers produced by CD-NTases that are increasingly implicated in a wide range of CBASS antiviral defense systems in bacteria and archaea [55,56,57,58,59,60,61]. Csa3 and SAVED domain proteins, for example, are likely to recognize overlapping subsets of these oligonucleotide second messengers.

## Figures and Tables

**Table 1:** Data collection.

Dataset	SsCsa3-cA_4_
Wavelength (Å)	1.54
Space Group	P2_1_2_1_2_1_
Cell Constants (a,b,c; Å) (α, β, γ; ^o^)	46.1, 93.4, 105.2 90, 90, 90
Resolution Range ^a^ (Å)	27.91–2.45 (2.49–2.45)
Unique Reflections ^a^	16,551
Average Redundancy ^a^	10.4 (10.4)
I/σ ^a^	36.4 (2.5)
Completeness (%)	96.8(100)
R_sym_ ^a,b^ (%)	7.9 (82.0)
R_pim_ ^a^ (%)	2.6 (26.5)
CC1/2 ^a^	0.99 (0.737)

^a^ Numbers in parenthesis refer to the highest resolution shell. ^b^ Rsym=100 *ΣhΣi|Ii (h)-<I (h)>|/ΣhI (h) where Ii (h) is the ith measurement of reflection h and <I (h)> is the average value of the reflection intensity.

**Table 2:** Model refinement.

Model Refinement	
R_work_ ^c^ (%)	20.3 (28.8)
R_free_ ^c^ (%)	24.3 (34.6)
Real Space CC ^d^ (%)	84.8
Mean B Value (overall; Å^2^)	85.0
Coordinate Error (based on maximum likelihood, Å)	0.37
RMSD from Ideality:	
Bonds (Å)	0.005
Angles (°)	0.687
Ramachandran Plot ^e^	
Most Favored (%)	96.0
Additional Allowed (%)	4.0
PDB Accession Code	6W11

^c^ Rwork = Σ||Fo|-Fc||/ΣFo| where Fo and Fc are the observed and calculated structure factor amplitudes used in refinement. Rfree is calculated as Rwork, but using the “test” set of structure factor amplitudes that were withheld from refinement (4.9%). ^d^ Correlation coefficient (CC) is agreement between the model and 2mFo-DFc electron density map. ^e^ Calculated using MolProbity (35).

## Data Availability

Coordinates and structure factors have been deposited in the protein databank with accession number 6W11 (https://www.rcsb.org/structure/6W11, deposited on: 3 March 2020).

## Supplementary Materials

The following are available online at https://www.mdpi.com/article/10.3390/biom11121852/s1, Figure S1: CRISPR systems in the *Saccharolobus solfataricus* P2 genome, Figure S2: cA_4_ omit map, Figure S3: Nonspecific binding to the I-A (1) cas4a promoter and CRISPER locus F leader, Figure S4: Nonspecific binding to CRISPR loci B and E leaders, Figure S5: Nonspecific binding to the CRISPR locus F leader and I-A (1) cas1 promoter, Table S1: ITC values, Table S2: PCR primers for EMSA fragments, Table S3: Fluorescence polarization constructs, Table S4: Acquisition assay PCR primers, Table S5: Acquisition cassette synteny, Table S6: Correlation of Csa3a and CAPPa, Table S7: RNA conformers for cA_4_, Video S1: cA_4_-Induced conformational change, Video S2: Conformational change and DNA recognition.
