# Supplementary material for: Cyclic Tetra-Adenylate (cA4) Recognition by Csa3; Implications for an Integrated Class 1 CRISPR-Cas Immune Response in Saccharolobus solfataricus
Source: Biomolecules. 2021 Dec 9;11(12):1852. doi: 10.3390/biom11121852 (PMC8699464; doi:10.3390/biom11121852)
Supplement: Supplementary file 1 [file biomolecules-11-01852-s001.zip › Biomolecules_RevisedSupplemental.pdf]

Supporting Online Materials For:

Cyclic Tetra-Adenylate (cA_4) Recognition by Csa3; Implications for an Integrated Class 1 CRISPR-Cas Immune Response

Alexander A. Charbonneau[1,2], Debra M. Eckert[3], Colin C. Gauvin[1,2], Nathaniel G. Lintner[§], and C. Martin

[1] Department of Chemistry and Biochemistry, Montana State University, Bozeman, MT 59717 USA

[2] Thermal Biology Institute, Montana State University, Bozeman, MT 59717 USA

[3] University of Utah School of Medicine, Salt Lake City, UT, USA

[§] Current address: Medicine Design, Pfizer, Cambridge, MA 02139

* Correspondence: lawrence@montana.edu; Tel.: +1 406-994-5382

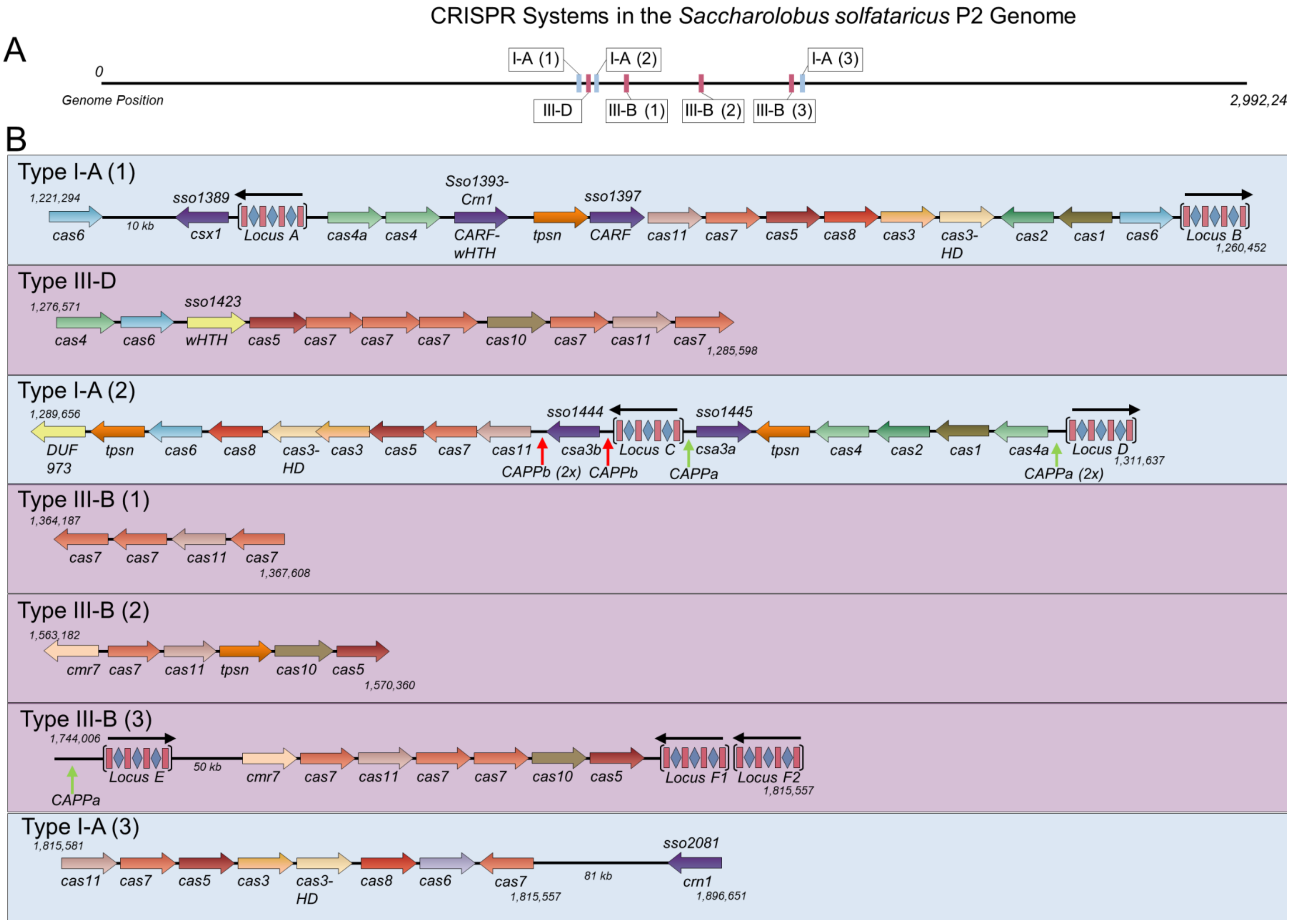
CRISPR Systems in the Saccharolobus solfataricus P2 Genome
A
0
Genome Position
2,992,24
I-A (1)
I-A (2)
I-A (3)
III-D
III-B (1)
III-B (2)
III-B (3)
B
Type I-A (1)
1,221,294
cas6
10 kb
sso1389
csx1
Locus A
cas4a
cas4
Sso1393-
Crn1
CARF-
wHTH
tpsn
sso1397
CARF
cas11
cas7
cas5
cas8
cas3
cas3-
HD
cas2
cas1
cas6
Locus B
1,260,452
Type III-D
1,276,571
cas4
cas6
sso1423
wHTH
cas5
cas7
cas7
cas7
cas10
cas7
cas11
cas7
1,285,598
Type I-A (2)
1,289,656
DUF
973
tpsn
cas6
cas8
cas3-
HD
cas3
cas5
cas7
cas11
CAPPb (2x)
sso1444
csa3b
CAPPb
Locus C
sso1445
csa3a
CAPPa
tpsn
cas4
cas2
cas1
cas4a
CAPPa (2x)
Locus D
1,311,637
Type III-B (1)
1,364,187
cas7
cas7
cas11
cas7
1,367,608
Type III-B (2)
1,563,182
cmr7
cas7
cas11
tpsn
cas10
cas5
1,570,360
Type III-B (3)
1,744,006
CAPPa
Locus E
50 kb
cmr7
cas7
cas11
cas7
cas7
cas10
cas5
Locus F1
Locus F2
1,815,557
Type I-A (3)
1,815,581
cas11
cas7
cas5
cas3
cas3-
HD
cas8
cas6
cas7
1,815,557
81 kb
sso2081
crn1
1,896,651

Supplementary Figure S1. Overview of CRISPR/Cas elements in *S. solfataricus* P2. **A**) General locations of the CRISPR/Cas clusters are represented by blue (Type I clusters) and red (Type III) clusters) bars on a black line representing the ~3 Mbp genome. **B**) Genes are colored based on the encoded gene. The acquisition genes, *cas1, cas2, cas4, cas4a* are in shades of green and *cas6* (crRNA maturation) is in blue. The interference genes are in shades of red to yellow. CARF domain genes are violet. Non-*cas* genes or genes of unknown function are colored bright yellow, and transposons are colored bright orange. CRISPR loci are depicted as repeated red bars (repeats) and blue diamonds (spacers). The positions of CAPPa are indicated by green vertical arrows. The locations of an unrelated binding motif reported for Csa3b [1], which we denote CAPPb, are also indicated as vertical red arrows. Italic numbers at either end of the clusters denote genome positions.

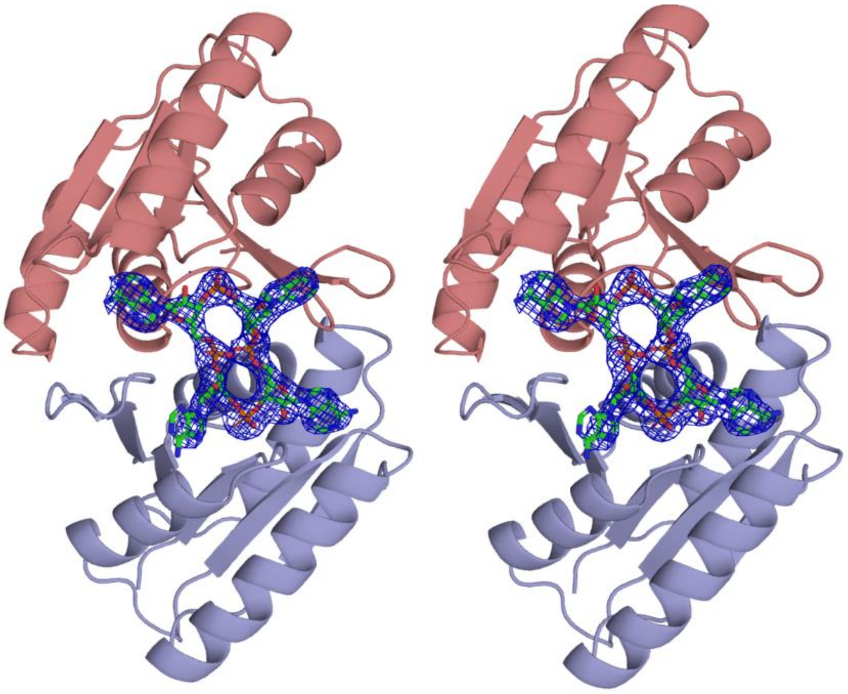

Supplemental Figure S2. cA_4 omit map. Ribbon diagram of the Csa3 dimer bound to cA_4. Chain A is blue and chain B is red. cA_4 is in a stick representation with C atoms in green. The $2F_o$-F_c electron density omit map contoured at 1.0σ (blue) was generated with a single round of refinement with Csa3 without cA_4.

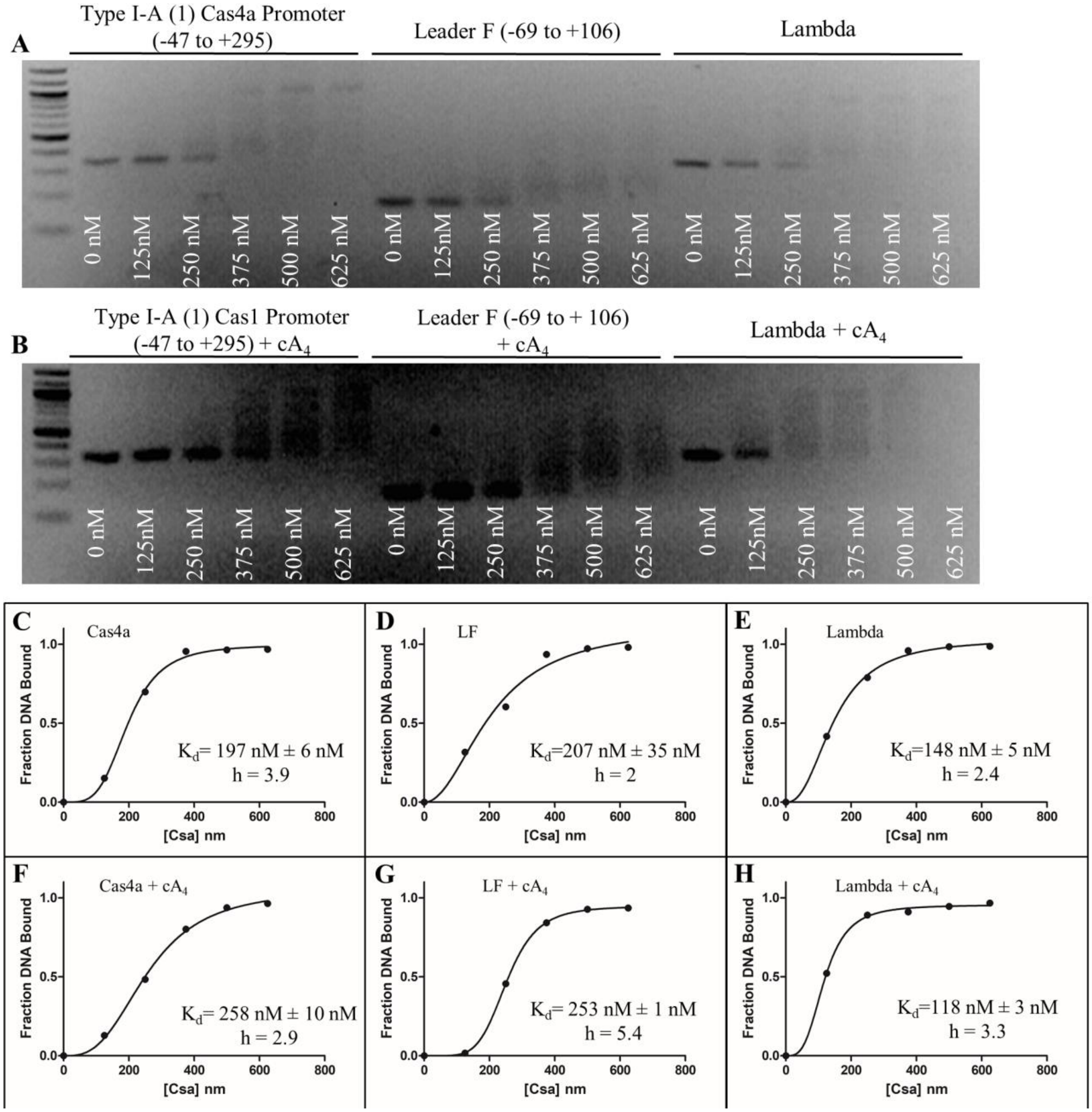

Supplemental Figure S3. Nonspecific binding to the I-A (1) cas4a promoter and CRISPER locus F leader. **A**) Csa3 binds all fragments with ~200 nm affinity, including Lambda phage DNA indicating a nonspecific interaction in the absence of cA_4. **B**) Addition of cA_4 does not affect binding affinity or specificity. EMSA experiments were performed with 18 nM DNA and increasing [Csa3] dimer (denoted below the lanes). EMSA fragments are detailed in Supplemental Table S4. Lambda phage DNA was used as a negative control. **C-H.** Graphs generated in GraphPad Prism display curves for specific binding with a Hill coefficient (h) fit to binding data generated using ImageJ.

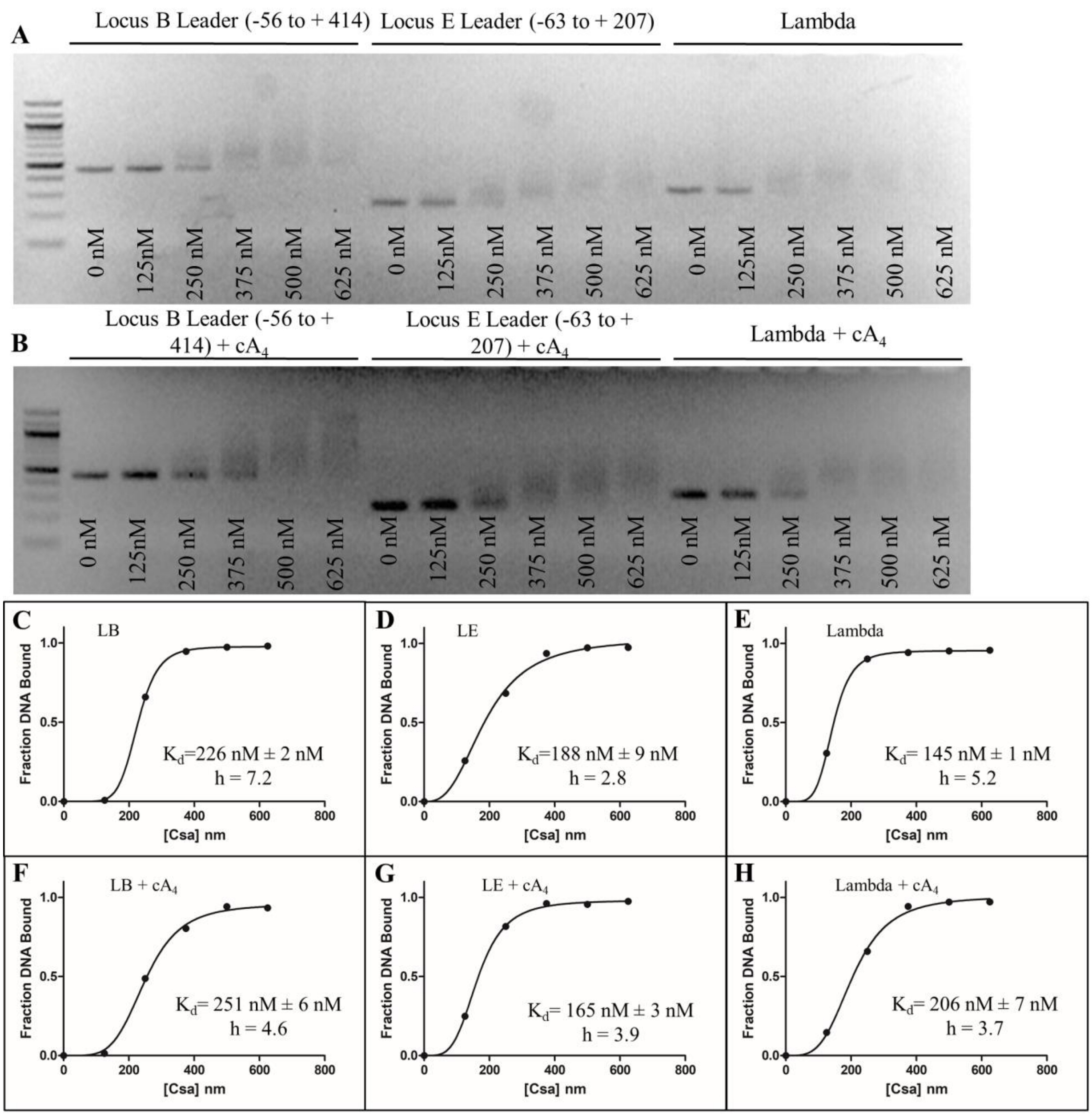

Supplemental Figure S4. Nonspecific binding to CRISPR loci B and E leaders. **A**) Csa3 binds all fragments with ~200 nm affinity, including Lambda phage DNA indicating a nonspecific interaction in the absence of cA_4. **B**) Addition of cA_4 does not affect binding affinity or specificity. EMSA experiments were performed with 18 nM DNA and increasing [Csa3] dimer (denoted below the lanes). EMSA fragments are detailed in Supplemental Table S4. Lambda phage DNA was used as a negative control. **C-H.** Graphs generated in GraphPad Prism display curves for specific binding with a Hill coefficient (h) fit to binding data generated using ImageJ.

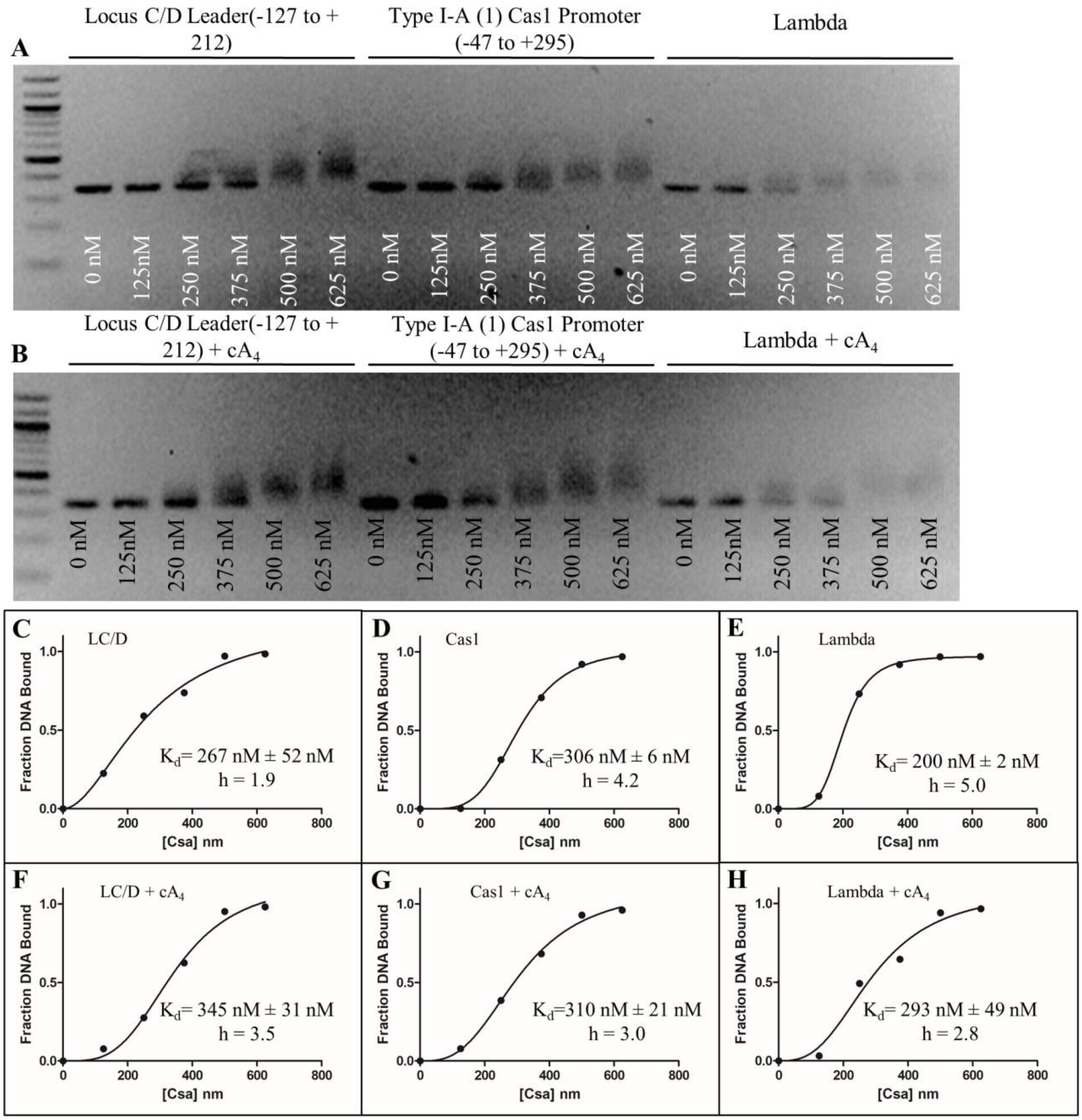

Supplemental Figure S5. Nonspecific binding to the CRISPR locus F leader and I-A(1) cas1 promoter. **A**) Csa3 binds all fragments with ~200-300 nm affinity, including Lambda phage DNA indicating a nonspecific interaction in the absence of cA_4. **B**) Addition of cA_4 does not affect binding affinity or specificity. EMSA experiments were performed with 18 nM DNA and increasing [Csa3] dimer (denoted below the lanes). EMSA fragments are detailed in Supplemental Table S4. Lambda phage DNA was used as a negative control. **C-H.** Graphs generated in GraphPad Prism display curves for specific binding with a Hill coefficient (h) fit to binding data generated using ImageJ.

Supplemental Tables

Supplemental Table S1 – ITC Values

[cA4] (μM)	K_D (μM)*	$\Delta H_{25°C}$ (Cal/mol)*	$\Delta S_{25°C}$ (Cal/mol/deg)*	N*†
250	1.09 (0.10)	13,490 (390)	72.5 (1.2)	0.546 (0.028)
500	1.48 (0.19)	13,380 (470)	71.6 (1.3)	0.608 (0.017)

*Duplicate ITC runs were averaged, with the range indicated.
†Stoichiometry of ligand:protein interaction relative to the Csa3a dimer.

Supplemental Table S2 - PCR Primers For EMSA Fragments

Fragment Name	Annotation	Size (bp)	Genome Location	Annealing Temp. °C	Primers (5' to 3') - Forward (top), Reverse (bottom)
Cas4a (II)	Promoter region for Cas4a (Group 2 acquisition cassette)	350	1305123-1305472	63	ATAACGTCTAGGCTTTATCGGAGG<br>CGTTTGATGTTAGTTTCTTAGGAGGTA
Cas4a (I)	Promoter region for Cas4a/Cas4 (Group 1 acquisition cassette)	342	1240163-1240504	63	CCGCAACTAACCCGTTCC<br>TTTATCCTCTTTGAAAGCAACAGC
Cas1	Promoter region for Cas1/Cas2 (Group 1 acquisition cassette)	350	1253317-1253666	63	GAATGGAGTTCTGTAGGGGCAA<br>TAATCGAAATGTAAGTGTGTAAATCACAG
LB	Promoter region directly upstream of CRISPR Leader B	470	1260396-1260865	63	GATATTATACCGGTTGAGCCTGC<br>CAGCCTTAGTGAAATTAGGAAATATTACG
LC/D	Promoter region directly upstream of CRISPR Leaders C/D	339	1305422-1305760	63	TGTCCCGTTTTTGTAAGTGGG<br>TCGATAGAAAGATTTAAATATGCGGAATAC
LE	Promoter region directly upstream of CRISPR Leader E	270	1743799-1744068	64	GGGAAAGAGTTCCCCCGTA<br>ACGTCGCTGCCAATTTTCC
LF	Promoter region directly upstream of CRISPR Leader F	175	1811222-1811396	62	CTGTTAGTAAGCCCCTTGCTAC<br>AGAGAATACTTGTTACCTAACTGGT
Lambda	Control Fragment from Lambda phage genome	350	24398-24747	62	TAGGAATTGGTTAGCAAGTTACTACC<br>TTGCTAACAGGTATCGTTTGGAG

Supplemental Table S2. PCR primers used to generate EMSA fragments. The fragment name denotes the name used to reference the indicated fragment in experimental procedures and results. The genome location indicates the location of the amplified fragment within the genome of *S. solfataricus* P2 for all fragments besides Lambda-350 which was generated from the Lambda phage genome.

Supplemental Table S3 - Fluorescence Polarization DNA Constructs

Fragment Name	Size (bp)	*S. solfataricus* P2 Genome Location	Annealing Temp. °C	ssDNA Oligonucleotides or Primers (5' to 3')
20bp 5'-FAM I-A(2) Cas4a CAPPa	20	1305298-1305317	N/A	/56-FAM/TTCCCGAATTTACTAGGGAT<br>AAGGGCTTAAATGATCCCTA
28bp 5'-FAM Idealized CAPPa	28	N/A	N/A	/56-FAM/GTTAATCCCGAATTAATTCGGGATTAAC<br>(self-complimentary)
75bp 5'-FAM I-A(2) Cas4a CAPPa	75	1305298-1305372	61	/56-FAM/TTCCCGAATTTACTAGGGAT<br>TCCCTAGTTTAATTATTAATCTTTATATAGAGATGA

Supplemental Table S**4** - Acquisition Assay PCR Primers

Fragment Name	Size (bp)	*S. solfataricus* P1 Genome Location	Annealing Temp. °C	Primers (5' to 3') - Forward (top), Reverse (bottom)
Locus A	147	1321505-1321651	57.5	ACTAAAATTGGTCGCATGAAGAGTA<br>ACTTAGCAGAATATCTACTGAAATATCTGC
Locus C	147	1384343-1384489	57.6	AACACTCCCGTACCAATTTCTTATT<br>CTAACACAATCCGATTAAAGTATATCCTCA
Locus D	170	1392982-1393151	62	CAGAGAAAAGCTTATAAATAACTAAGGAGA<br>GGTCGACAATCCCGTAGATC
Locus E	152	1817367-1817518	56.3	GGAAAACCCGGGAATAGTAAAAA<br>TGCCAATTTTCCGCCAATTC
Locus F	188	1883103-1883290	54.8	CCTGATTTATTCAATCCAGAGAATTT<br>GGCAATAGTGAATGGTGTAGTTATA
Locus F2	156	1884684-1884839	57.7	TAGTAAGCCCCTTGCTACAACT<br>CCTAACTGGTTGAGCAAAAACTTTAAATAT

Supplemental Table S5 – Acquisition Cassette Synteny

Strain																			
A. brierleyi DSM 1651	*csa3b*	-//-	< Locus			CAPPa			*cas4a >*	*cas1 >*	*cas2 >*		*cas4 >*	*< csa3a*	CAPPa	Locus >			
A. hospitalis W1		*< csa3b*	< Locus			CAPPa			*cas4a >*	*cas1 >*	*cas2 >*	CAPPa	*cas4 >*	*< csa3a*	CAPPa	Locus >			
A. sulfidivorans JP7		*< csa3b*	< Locus		CAPPa	CAPPa			*cas4a >*	*cas1 >*	*cas2 >*		*cas4 >*	*< csa3a*	CAPPa	Locus >			
M. prunae Ron 12		*< csa3b*	< Locus	CAPPa	CAPPa	CAPPa			*cas4a >*	*cas1 >*	*cas2 >*		*cas4 >*	Csa3a >	CAPPa	Locus >			
M. sedula ARS120-1		*< csa3b*	< Locus	CAPPa	CAPPa	CAPPa			*cas4a >*	*cas1 >*	*cas2 >*		*cas4 >*	Csa3a >	CAPPa	Locus >			
M. sedula ARS120-2		*< csa3b*	< Locus	CAPPa	CAPPa	CAPPa			*cas4a >*	*cas1 >*	*cas2 >*		*cas4 >*	Csa3a >	CAPPa	Locus >			
M. sedula ARS50-1		*< csa3b*	< Locus	CAPPa	CAPPa	CAPPa			*cas4a >*	*cas1 >*	*cas2 >*		*cas4 >*	Csa3a >	CAPPa	Locus >			
M. sedula ARS50-2		*< csa3b*	< Locus	CAPPa	CAPPa	CAPPa			*cas4a >*	*cas1 >*	*cas2 >*		*cas4 >*	Csa3a >	CAPPa	Locus >			
M. sedula DSM 5348		*< csa3b*	< Locus	CAPPa	CAPPa	CAPPa			*cas4a >*	*cas1 >*	*cas2 >*		*cas4 >*	Csa3a >	CAPPa	Locus >			
M. sedula SARC-M1		*< csa3b*	< Locus	CAPPa	CAPPa	CAPPa			*cas4a >*	*cas1 >*	*cas2 >*		*cas4 >*	Csa3a >	CAPPa	Locus >			
M. tengchongensis Ric-A			< Locus			CAPPa			*cas4a >*	*cas1 >*	*cas2 >*		*cas4 >*	*< csa3a*	CAPPa	Locus >			
S. solfataricus P1			< Locus		CAPPa	CAPPa			*cas4a >*	*cas1 >*	*cas2 >*		*cas4 >*	*< csa3a*	CAPPa	Locus >	*csa3b >*		
S. solfataricus P2			< Locus		CAPPa	CAPPa			*cas4a >*	*cas1 >*	*cas2 >*		*cas4 >*	*< csa3a*	CAPPa	Locus >	*csa3b >*		
S. solfataricus POZ149			< Locus		CAPPa	CAPPa			*cas4a >*	*cas1 >*	*cas2 >*		*cas4 >*	*< csa3a*	CAPPa	Locus >	*csa3b >*		
S. sp. E-5-1-F			< Locus		CAPPa	CAPPa			*cas4a >*	*cas1 >*	*cas2 >*		*cas4 >*	*< csa3a*	CAPPa	Locus >	-//-	*csa3b*	
S. sp. S-194			< Locus			CAPPa	*< csa3a*	CAPPa	*cas4a >*	*cas1 >*	*cas2 >*		*cas4 >*		CAPPa	Locus >			
S. islandicus HVE10-4		*< csa3b*	< Locus			CAPPa			*cas4a >*	*cas1 >*	*cas2*/CAPPa		*cas4 >*	*< csa3a*	CAPPa	Locus >			
S. islandicus L.S.2.15			< Locus			CAPPa			*cas4a >*	*cas1 >*	*cas2 >*		*cas4 >*	*< csa3a*	CAPPa	Locus >	*csa3b >*		
S. islandicus LAL14/1			< Locus			CAPPa			*cas4a >*	*cas1 >*	*cas2*/CAPPa		*cas4 >*	*< csa3a*	CAPPa	Locus >	*csa3b >*		
S. islandicus M.14.25		*< csa3b*	< Locus		CAPPa	CAPPa			*cas4a >*	*cas1 >*	*cas2 >*		*cas4 >*	*< csa3a*	CAPPa	Locus >			
S. islandicus M.16.27			< Locus		CAPPa	CAPPa			*cas4a >*	*cas1 >*	*cas2 >*		*cas4 >*	*< csa3a*	CAPPa	Locus >	*csa3b >*		
S. islandicus REY15A			< Locus			CAPPa			*cas4a >*	*cas1 >*	*cas2*/CAPPa		*cas4 >*	*< csa3a*	CAPPa	Locus >	*csa3b >*		
S. islandicus Y.G.57.14		*< csa3b*	< Locus			CAPPa			*cas4a >*	*cas1 >*	*cas2*/CAPPa		*cas4 >*	*< csa3a*	CAPPa	Locus >			
S. islandicus YN.15.51			< Locus			CAPPa			*cas4a >*	*cas1 >*	*cas2*/CAPPa		*cas4 >*	*< csa3a*	CAPPa	Locus >	*csa3b >*		
S. tokodaii 7			< Locus		CAPPa	CAPPa	*< csa3a*	CAPPa	*cas4a >*	*cas1 >*	*cas2 >*		*cas4 >*			< Locus		CAPPa	CAPPa
S. ohwakuensis TA-1	*csa3b*	-//-	< Locus		CAPPa	CAPPa	*< csa3a*	CAPPa	*cas4a >*	*cas1 >*	*cas2 >*		*cas4 >*			< Locus		CAPPa	CAPPa

*single nucleotide mutation

Supplemental Table S5. Acquisition cassette synteny for Sulfolobales containing Csa3a. The csa3a gene is generally associated with the acquisition cassette (green genes), and a CAPPa sequence is also present immediately upstream of the cas4a gene. Gene and locus transcript direction are indicated by the adjacent arrows. Gene positions are relative, and transposases or proteins of unknown function are not shown. The -//- symbolizes large genomic distance between genes.

Supplemental Table S6 – Correlation of Csa3a and CAPPa

Strain	aCas	Csa3a	CAPPa
Acidianus ambivalens LEI 10	-	-	-
Acidianus brierleyi DSM 1651	+	+	+
Acidianus hospitalis W1	+	+	+
Acidianus manzaensis YN-25	-	-	-
Acidianus sulfidivorans JP7	+	+	+
Metallosphaera cuprina Ar-4	-	-	-
Metallosphaera hakonensis JCM 8857	-	-	-
Metallosphaera prunae Ron 12	+	+	+
Metallosphaera sedula ARS120-1	+	+	+
Metallosphaera sedula ARS120-2	+	+	+
Metallosphaera sedula ARS50-1	+	+	+
Metallosphaera sedula ARS50-2	+	+	+
Metallosphaera sedula DSM 5348	+	+	+
Metallosphaera sedula SARC-M1	+	+	+
Metallosphaera tengchongensis Ric-A	+	+	+
Saccharolobus solfataricus P1	+	+	+
Saccharolobus solfataricus P2	+	+	+
Saccharolobus solfataricus POZ149	+	+	+
Saccharolobus solfataricus 98/2*	+	-	+
Stygiolobus azoricus FC6	-	-	-
Sulfodiicoccus acidiphilus HS-1	+	-	-
Sulfolobus acidocaldarius DG1	-	-	-
Sulfolobus acidocaldarius DSM 639	-	-	-
Sulfolobus acidocaldarius N8	-	-	-
Sulfolobus acidocaldarius Ron12/I	-	-	-
Sulfolobus acidocaldarius SUSAZ	-	-	-
Sulfolobus acidocaldarius Y14 13-1	-	-	-
Sulfolobus acidocaldarius Y14 16-22	-	-	-
Sulfolobus acidocaldarius Y14 18-5	-	-	-
Sulfolobus acidocaldarius Y14 20-20	-	-	-
Sulfolobus islandicus HVE10/4	+	+	+
Sulfolobus islandicus L.S.2.15	+	+	+
Sulfolobus islandicus LAL14/1	+	+	+
Sulfolobus islandicus M.14.25	+	+	+
Sulfolobus islandicus M.16.27	+	+	+
Sulfolobus islandicus M.16.4	+	**	+
Sulfolobus islandicus REY15A	+	+	+
Sulfolobus islandicus Y.G.57.14	+	+	+
Sulfolobus islandicus Y.N.15.51	+	+	+
Sulfolobus sp. A20	-	-	-
Sulfolobus sp. E11-6	-	-	-
Sulfolobus sp. E5-1-F	+	+	+
Sulfolobus sp. S-194	+	+	+
Sulfolobus tokodaii 7	+	+	+
Sulfuracidifex tepidarius IC-006	-	-	-
Sulfuracidifex tepidarius IC-007	-	-	-
Sulfurisphaera ohwakuensis TA-1	+	+	+

Supplemental Table S6. Correlation of Csa3a and CApPa. Csa3 presence or absence correlates strongly with the presence or absence of CAPPa in the acquisition cassette (aCas). A "+" denotes the presence of the indicated element, "-" denotes absence, "*" indicates combined parent and daughter strains and "**" indicates viral insertion into csa3a gene. csa3a is present in all sequenced genomes with CAPPa except those derived from *S. solfataricus* 98/2.

Supplemental Table S7 - RNA Conformers for cA_4*

Nucleotide	δ,δ,γ	Name	Suite	δ-1	ε	ζ	α	β	γ	δ
A1	22m	2o	2'emmtm2'	158.08	-145.90	50.51	-	-119.33	-71.42	156.24
A2	22p	6p	2'epptp2'	156.24	-151.05	125.27	61.82	167.29	49.25	155.06
A3	22m	2o	2'emmtm2'	155.06	-146.90	75.04	70.37	165.48	-66.01	153.03
A4	22p	6p	2'epptp2'	153.03	-	-	74.01	-172.64	55	158.08

*For RNA backbone conformer nomenclature see Richardson et al. [2].

Supplemental Videos

Supplemental Video S1. cA_4 Induced Conformational Change.

Supplemental Video S2. Conformational Change and DNA Recognition.

Supplemental References

1. He, F.; Vestergaard, G.; Peng, W.; She, Q.; Peng, X., CRISPR-Cas type I-A Cascade complex couples viral infection surveillance to host transcriptional regulation in the dependence of Csa3b. *Nucleic Acids Res* **2017,** *45* (4), 1902-1913.
2. Richardson, J. S.; Schneider, B.; Murray, L. W.; Kapral, G. J.; Immormino, R. M.; Headd, J. J.; Richardson, D. C.; Ham, D.; Hershkovits, E.; Williams, L. D.; Keating, K. S.; Pyle, A. M.; Micallef, D.; Westbrook, J.; Berman, H. M.; Consortium, R. N. A. O., RNA backbone: consensus all-angle conformers and modular string nomenclature (an RNA Ontology Consortium contribution). *RNA* **2008,** *14* (3), 465-81.
